# Proteomic analysis of the actin cortex in interphase and mitosis

**DOI:** 10.1242/jcs.259993

**Published:** 2022-08-26

**Authors:** Neza Vadnjal, Sami Nourreddine, Geneviève Lavoie, Murielle Serres, Philippe P. Roux, Ewa K. Paluch

**Affiliations:** 1 Laboratory for Molecular Cell Biology, University College London, London WC1E 6BT, UK; 2 Department of Physiology, Development and Neuroscience, University of Cambridge, Cambridge CB2 3DY, UK; 3 Institute for Research in Immunology and Cancer, Université de Montréal, Montreal QC, H3T 1J4, Canada; 4 Department of Pathology and Cell Biology, Faculty of Medicine, Université de Montréal, Montreal, QC, H3T 1J4, Canada

**Keywords:** Actin, Cell cortex, Cell division, Cortical tension, Mass spectrometry, Septin

## Abstract

Many animal cell shape changes are driven by gradients in the contractile tension of the actomyosin cortex, a thin cytoskeletal network supporting the plasma membrane. Elucidating cortical tension control is thus essential for understanding cell morphogenesis. Increasing evidence shows that alongside myosin activity, actin network organisation and composition are key to cortex tension regulation. However, owing to a poor understanding of how cortex composition changes when tension changes, which cortical components are important remains unclear. In this article, we compared cortices from cells with low and high cortex tensions. We purified cortex-enriched fractions from cells in interphase and mitosis, as mitosis is characterised by high cortical tension. Mass spectrometry analysis identified 922 proteins consistently represented in both interphase and mitotic cortices. Focusing on actin-related proteins narrowed down the list to 238 candidate regulators of the mitotic cortical tension increase. Among these candidates, we found that there is a role for septins in mitotic cell rounding control. Overall, our study provides a comprehensive dataset of candidate cortex regulators, paving the way for systematic investigations of the regulation of cell surface mechanics.

This article has an associated First Person interview with the first author of the paper.

## Abstract (precis)

**Summary:** Contractile tension at the actomyosin cortex is a key determinant of cell shape. Cortices from cells with high and low tension were analysed using mass spectrometry, generating a dataset of candidate cortex mechanics regulators.

## INTRODUCTION

Changes in animal cell shape are largely controlled by the actin cytoskeleton. In particular, the actomyosin cortex, a thin (∼150 nm thick) network of actin, myosin and associated proteins underneath the plasma membrane, drives contraction-based cellular deformations (reviewed in Salbreux et al., 2012; Chugh and Paluch, 2018). Contractile tension is generated in the cortex by myosin motors pulling on actin filaments, and increasing cortical tension promotes cell rounding. For instance, a strong increase in cortical tension at the onset of mitosis typically leads cells to round up in prometaphase (Stewart et al., 2011; Cadart et al., 2014). The resulting mitotic rounded shape provides the space required for spindle assembly, positioning, and accurate chromosome separation (reviewed in Lancaster and Baum, 2014). Furthermore, gradients in cortical tension lead to local contractions and induce cell shape changes driving processes such as cell division, cell migration and epithelial morphogenesis (reviewed in Levayer and Lecuit, 2012; Chugh and Paluch, 2018). Given its importance in the regulation of cell shape, it is thus essential to systematically identify the key factors controlling cortical tension.

The role of myosin motors in cortical tension generation has been extensively characterised (Tinevez et al., 2009; Murrell et al., 2015; Ramanathan et al., 2015; Truong Quang et al., 2021). However, a number of recent studies have highlighted the importance of other actin-binding proteins. In particular, regulators of actin filament nucleation and length, as well as crosslinkers, have been shown to play a role in the control of cortical tension (Bergert et al., 2012; Chugh et al., 2017; Ding et al., 2017; reviewed in Koenderink and Paluch, 2018). Furthermore, intermediate filaments have been shown to contribute to the cortical tension increase observed in mitotic cells (Duarte et al., 2019; Serres et al., 2020). Taken together, the emerging view is that cortical tension is controlled by a variety of mechanisms that modulate myosin activity and actin network organisation, as well as cortex interactions with other cytoskeletal networks. However, systematic investigations of cortex tension regulation have been significantly hindered by our limited understanding of how cortical composition changes when tension changes.

In this Tools and Resources article, we set out to systematically investigate which actin-related proteins display different levels at the cortex in cells with high and low cortical tension. To this aim, we analysed the protein composition of actin cortices in cells synchronised in interphase and mitosis, because mitotic cells display considerably higher cortical tension compared to interphase cells (Chugh et al., 2017). We used purified cellular blebs, as we have previously shown that blebs assemble a cortex similar to the cellular cortex, and can be isolated from cells, yielding cortex-enriched cellular fractions (Biro et al., 2013; Bovellan et al., 2014). We compared, using mass spectrometry, the composition of blebs separated from interphase and mitotic cells. We identified 922 different proteins consistently present in blebs from both mitotic and interphase cells, among which 238 were actin-related proteins. Finally, we investigated one of the candidates identified, the septin family of proteins, and showed that septins regulate mitotic cell rounding, highlighting the potential of our dataset to identify new cortex and cell shape regulators. Taken together, our systematic analysis generates a list of candidate regulators of the cortical remodelling taking place at mitosis entry. It also identifies septins, and in particular septin 9, as important regulators of cortex-driven cell rounding at the transition between interphase and mitosis.

## RESULTS

### Isolation of actin cortex-enriched blebs from cells synchronised in interphase and mitosis

**Fig. 1.**
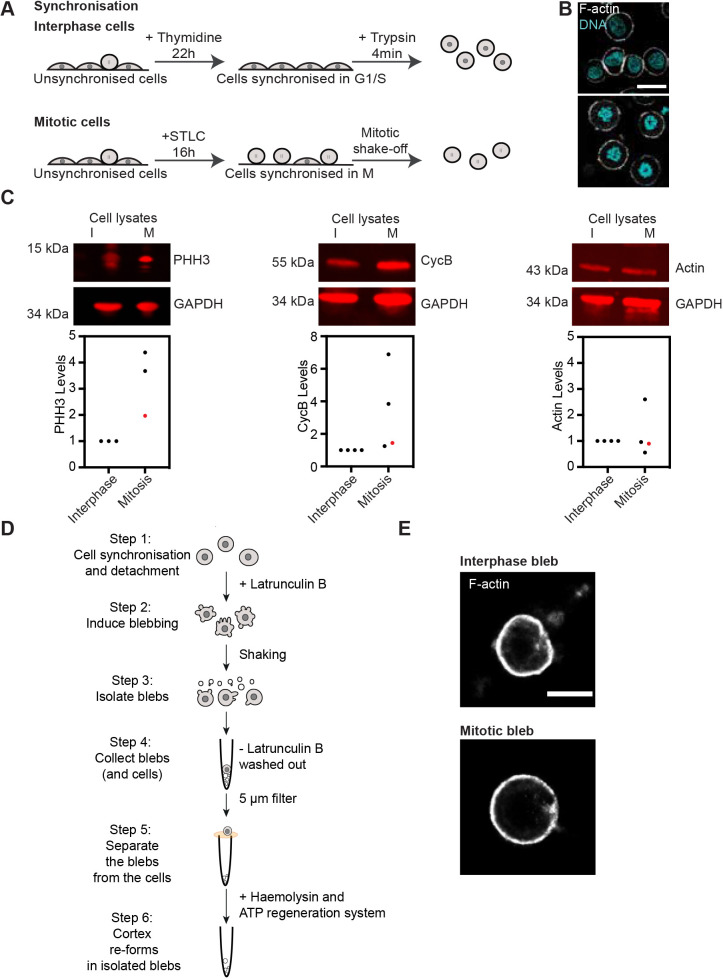
**Isolation of cortex-enriched blebs from interphase and mitotic cells.** (A) Schematic describing cell synchronisation in interphase (top) and mitosis (bottom). Prior to bleb isolation, cells were synchronised in interphase (G1/S phase) with a 22 h thymidine treatment, detached from the dish and rounded up using trypsin. For synchronisation in mitosis, the cell population was first enriched for mitotic cells with a 16 h treatment with the Eg5 inhibitor STLC, which prevents bipolar spindle formation, followed by a mitotic shake-off. (B) Representative confocal images of synchronised interphase (upper panel) and mitotic (lower panel) cells. Cyan, DAPI (DNA); white, phalloidin (F-actin). DAPI staining shows nuclear organisation of DNA in cells synchronised in interphase and condensed chromosomes in cells synchronised in mitosis. Scale bar: 20 µm. (C) Representative fluorescent western blots (upper panels), and related quantifications (lower panels) of mitotic markers [phosphorylated histone H3 (PHH3) and cyclin B] and actin levels (control) in interphase and mitotic cell lysates, confirming cell cycle phase synchronisation at the cell population level. Membranes are representative of *n*=4 samples used for quantification. GAPDH, loading control. For quantification, protein levels were normalised to the loading control (GAPDH) and interphase conditions. Red datapoints on graphs correspond to the samples represented in the images of western blot membranes (upper panels). (D) Schematic depicting the bleb isolation protocol. Blebs were isolated from round cells synchronised in interphase or mitosis (as described in A) (step 1). Blebbing was induced with treatment with the actin depolymerising drug latrunculin B (step 2). Blebs were detached from the cells with shear stress (step 3) and separated blebs were isolated from the cells using a 5 µm filter (steps 4, 5). Re-assembly of a dynamic actin cortex was induced in blebs through addition of a creatine phosphate-based ATP regeneration system and the alpha-toxin haemolysin, to permeabilise the bleb membranes to allow for ATP regeneration system uptake (step 6, see Materials and Methods for details). Step 1 was performed at 37°C and steps 2–6 were performed at room temperature. (E) Stochastic optical reconstruction microscopy (STORM) of the actin cortex in blebs isolated from interphase (upper panel) and mitotic (lower panel) cells. Isolated blebs were pre-treated with the ATP regeneration system (steps 5, 6 in D) prior to imaging. White, phalloidin (F-actin). Scale bar: 2 µm. Images in B and E are representative of three independent experiments.

We obtained synchronised rounded interphase (low cortex tension) and mitotic (high tension) cells using a protocol previously used in our laboratory (Chugh et al., 2017). Specifically, to enrich for cells in interphase, HeLa cells were synchronised in G1/S phase using thymidine (Fig. 1A, see Materials and Methods for details). Synchronised interphase cells were then detached and maintained in suspension (Fig. 1B, upper panel). To obtain mitotic cells, HeLa cells were synchronised in prometaphase using S-trityl-L-cystine (STLC). Mitotic cells were further enriched by mechanically separating them from adherent cells through a mitotic shake-off (Fig. 1B, bottom panel). We have previously shown that following this protocol, rounded interphase cells display a continuous cortex that can be compared to the cortex of mitotic cells, but display a cortical tension four-fold lower than mitotic cells, as measured by atomic force microscopy flat cantilever compression (Chugh et al., 2017). Finally, we checked synchronisation efficiency by DNA staining of the synchronised cell populations (Fig. 1B), and by immunoblotting for levels of the mitotic markers phosphorylated histone H3 and cyclin B (Fig. 1C). This confirmed that our synchronisation procedure yielded cell populations of predominantly rounded interphase and mitotic cells.

We then purified blebs to enrich for actin cortex components from synchronised rounded cells (Fig. 1D) by adapting a protocol we previously established (Biro et al., 2013; see Materials and Methods for details). In brief, blebbing was induced using the actin depolymerizing drug latrunculin B (Spector et al., 1983) and blebs were detached from cells by shear stress. We observed that mitotic HeLa cells yielded much fewer isolated blebs than the constitutively blebbing M2 cells used in previous studies (Biro et al., 2013; Bovellan et al., 2014). To account for this limited output, we considerably increased the amounts of cells used. Furthermore, in our protocol, blebs were isolated from non-adherent cells; we thus added a stringent filtration step to ensure the removal of entire cells from the cortex preparation (Fig. 1D). As previously described (Biro et al., 2013), latrunculin B was washed out (at Step 4, Fig. 1D), the bleb membrane was permeabilised with haemolysin A, and an exogeneous creatine phosphate-based ATP regeneration system was added to the buffer to facilitate cortex re-assembly (Step 6 in Fig. 1D, see Materials and Methods for details). We then imaged actin in samples of isolated blebs from each replicate prior to mass spectrometry. The blebs displayed a clearly defined actin cortex, confirming that the isolated blebs contain all the components required for successful cortex assembly (Fig. 1E; Fig. S1A). Furthermore, we confirmed that bleb lysates were enriched in actin and actin-binding proteins and displayed lower levels of nuclear proteins compared to what was found in whole-cell lysates (Fig. S1B). Taken together, our data show that this purification protocol successfully isolated cortical fractions from cells synchronised in interphase and mitosis.

### Proteomic analysis of actin cortex-enriched blebs from interphase and mitotic cells

**Fig. 2.**
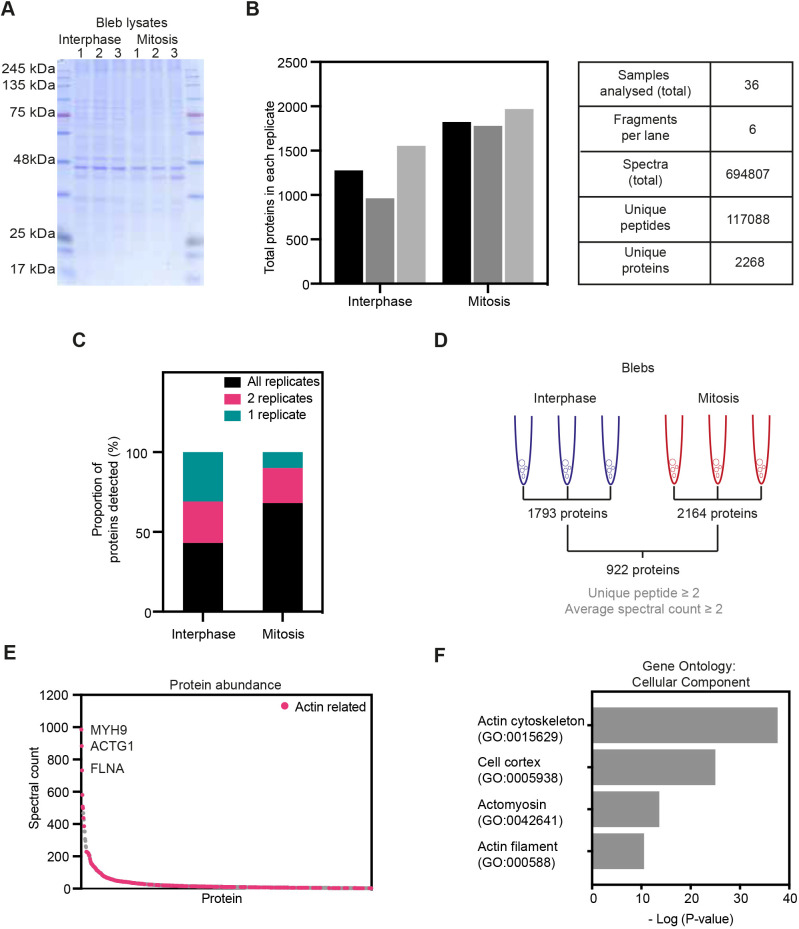
**Proteomic analysis of blebs isolated from interphase and mitotic cells.** (A) Coomassie staining of isolated blebs from interphase and mitosis in the three experimental replicates used for mass spectrometry. (B) Quantification of the number of proteins (left) and other overall readouts of the mass spectrometry analysis of samples from A. (C) Percentage of proteins detected in all, two or one of the replicates. (D) Schematic summarising the process of protein selection for further analysis. Mass spectrometry detected 1793 and 2164 different proteins in blebs isolated from interphase and mitotic cells, respectively. Out of these, 922 proteins with a unique peptide number above 2 and normalised spectral count above 2 were present in both interphase and mitotic blebs; these proteins were selected for further analysis. (E) Average normalised spectral counts in all (three per condition) replicates from interphase and mitotic blebs of the 922 proteins selected as described in D. Bright pink dots, actin-related proteins. The proteins with the highest average spectral count were myosin heavy chain IIA (MYH9), actin (ACTG1) and filamin A (FLNA). (F) Gene Ontology (GO) analysis of the selected 922 proteins, focusing on GO terms for ‘cellular components’ related to cell surface enrichment.

To identify proteins potentially involved in the regulation of cortical tension, we analysed blebs isolated from interphase and mitotic cells using liquid chromatography (LC)-tandem mass spectrometry (LC-MS/MS). For this, purified blebs from three biological replicates for each condition were lysed in denaturing Laemmli buffer and proteins were resolved by SDS-PAGE (Fig. 2A). Proteins were then subjected to in-gel trypsin digestion, and purified peptides were analysed by LC-MS/MS. Overall, we identified 117,088 unique peptides from 2268 unique proteins in interphase and mitosis (Fig. 2B,C; Table S1). To account for variations between experiments, the total number of spectra identified for each protein was normalised to the total spectral count detected within each replicate, using the first interphase replicate as a reference (see Materials and Methods for details). For further analysis, we only considered proteins that were detected from at least two unique peptides and with an average spectral count of two or higher in triplicates from both phases of the cell cycle, narrowing down the list to 922 proteins (Fig. 2D; Table S2). These criteria allowed us to focus on proteins that were consistently and reliably detected in our samples. Furthermore, focusing on proteins present in both interphase and mitosis eliminated multiple nuclear proteins that were detected at high levels in mitotic blebs but absent in interphase blebs (Table S1), likely due to increased cytoplasmic levels of nuclear components following nuclear envelope breakdown. Out of the 922 proteins selected, myosin heavy chain IIA (MYH9), actin (ACTG1) and filamin A (FLNA) were the most abundant based on spectral counts, which reflect both protein abundance and size (Fig. 2E). Of note, differentiating actin isoforms in mass spectrometry is challenging because of the high degree of conservation between isoforms (98.9% amino acid identity between ACTG1 and ACTB for instance), and resulting high degree of overlap between identified peptides. Thus, ACTG1 corresponds to various actin isoforms present in our samples. Overall, many of the most abundant proteins identified were actin-related proteins (Fig. 2E, bright pink dots).

To globally characterise the composition of the identified protein dataset, we next performed a Gene Ontology (GO) analysis for cellular components, molecular functions and biological processes (Fig. 2F; Fig. S1C,D). Cellular component GO terms describing actin filament (*P*=4.7×10^−11^), actomyosin (*P*=3.8×10^−14^), cell cortex (*P*=1.6×10^−25^) and actin cytoskeleton (*P*=3.7×10^−38^) were enriched for in our dataset (Fig. 2F). Analysis of molecular functions and biological processes GO terms further supports a high representation of actin-related proteins in isolated blebs (Fig. S1C,D). Taken together, our observations suggest that isolated blebs are indeed enriched in actin cortex components, and are thus a good model system to compare cortex composition between interphase and mitosis.

### Analysis of actin-related proteins in cortex-enriched blebs from interphase and mitotic cells

**Fig. 3.**
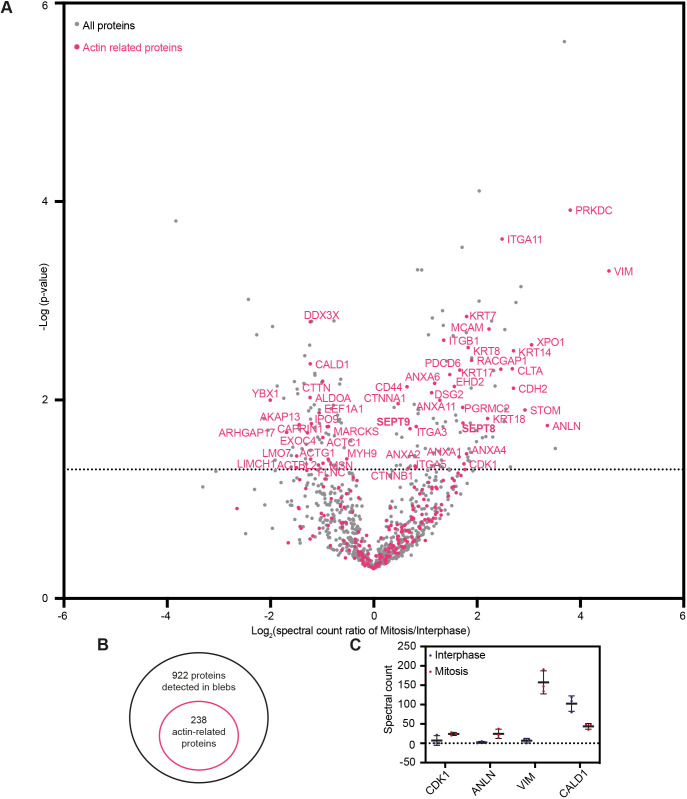
**Changes in cortical levels of actin-binding proteins between interphase and mitosis.** (A) Volcano plot of the 922 selected proteins detected in blebs, showing the enrichment (*x*-axis) and the significance of this enrichment (*P*-values, *y*-axis) between interphase and mitosis. Dotted line highlights −log_10_ (*P*-value)=1.3, corresponding to *P*-value=0.05; three independent replicates; statistics unpaired one-tailed Student's *t*-test. Bright pink dots: actin-related proteins. (B) Schematic of actin-related proteins among all proteins detected in blebs. (C) Spectral counts in the mass spectrometry analysis of mitotic and interphase blebs for proteins known to change in levels at the cortex between interphase and mitosis. Each data point corresponds to an individual replicate, with mean±s.d. shown. Spectral counts were normalised to account for variation between experiments.

To identify potential regulators of cortical tension, we next compared the levels of individual proteins detected in blebs isolated from cells in interphase and mitosis (Fig. 3A; Table S2). Only 180 out of the 922 proteins detected displayed a significant difference in protein levels between interphase and mitosis (corresponding to a *P*<0.05 data points above the dashed line in Fig. 3A), suggesting that cortex composition is largely similar between these two phases of the cell cycle. To narrow down our candidate list, we focused on actin-related proteins. These were selected based on a previously published list of F-actin-binding proteins identified by pull-down of F-actin-binding cellular fractions (Serres et al., 2020), complemented by manual curation to select further actin-related factors. Based on these criteria, we found 238 actin-related proteins in the cortex-enriched blebs (bright pink dots on Fig. 3A,B; Table S3). This narrowed down list included many proteins known to directly bind and regulate actin filaments, as well as membrane and adhesion proteins, the intermediate filament proteins vimentin and keratin, and various Rho-GTPases and their regulators. Notably, the other proteins found in blebs included tubulins and microtubule-binding proteins, as well as multiple factors involved in intracellular trafficking, which might also indirectly interact with the actin cortex (Tables S1, S2). Out of the 238 identified actin-related proteins, 54 significantly changed in levels between interphase and mitotic blebs to a *P*<0.05. Although this cut-off is somewhat arbitrary given the small number of experimental replicates inherent to a mass spectrometry study, this reduced list (Table 1) represents actin-related proteins that most consistently changed levels in cortex-enriched blebs between interphase and mitotic cells. Finally, we verified that there was a high degree of reproducibility between experimental replicates in our mass spectrometry analysis of the identified 54 proteins (Fig. S2A,B).

**Table 1.**
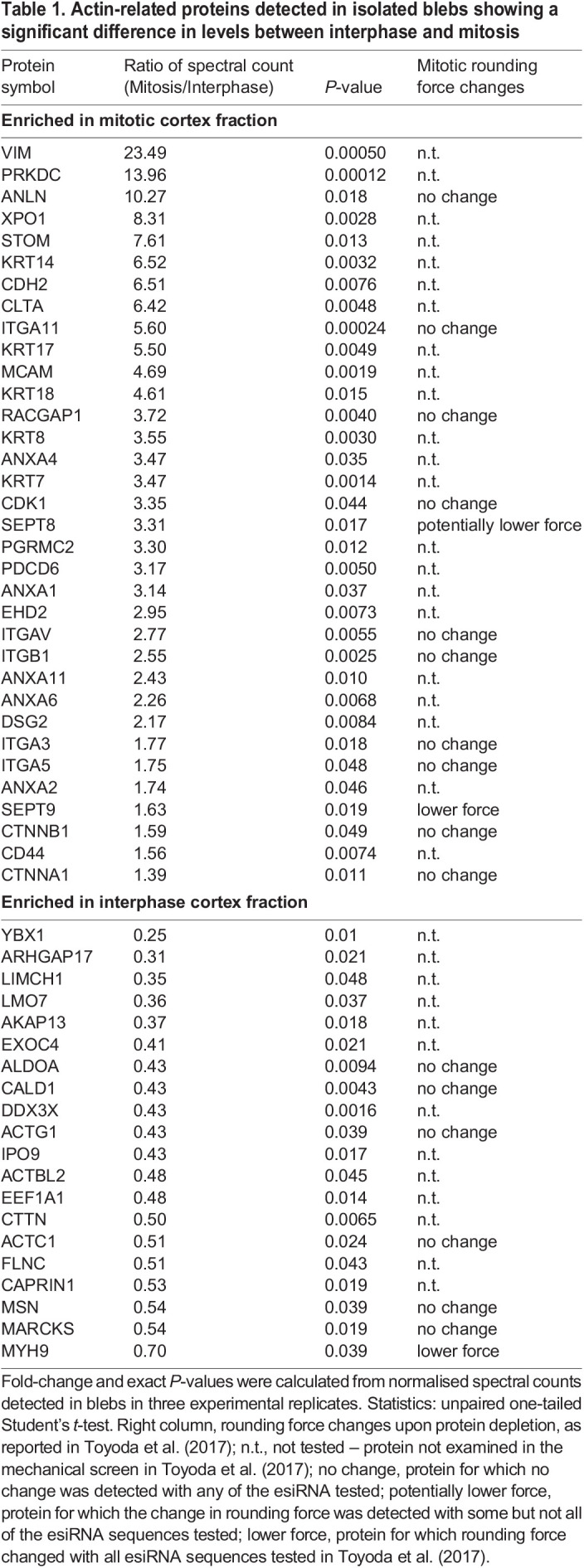
Actin-related proteins detected in isolated blebs showing a significant difference in levels between interphase and mitosis

Several of the identified proteins had previously been shown to change levels between interphase and mitosis (Fig. 3C). In particular, we found that the levels of cyclin-dependent kinase 1 (CDK1), a known cell cycle regulator that increases in mitosis (Lee and Nurse, 1987), and anillin, a cortical protein that translocates from the nucleus to the actin cortex in mitosis (Field and Alberts, 1995) were higher in mitotic compared to interphase blebs. Furthermore, the intermediate filament protein vimentin displayed strongly increased levels in mitotic blebs, consistent with recent findings demonstrating a role for vimentin in the mitotic cortex (Duarte et al., 2019; Serres et al., 2020). Finally, caldesmon displayed lower levels in mitotic compared to interphase blebs, consistent with previous observations showing that caldesmon dissociates from actin filaments during mitosis (Yamashiro et al., 1990). Together, these observations suggest that our mass spectrometry analysis successfully identifies proteins for which cortical levels change between interphase and mitosis.

We then compared the changes in bleb protein levels between interphase and mitosis to a published dataset reporting changes in protein levels in whole-cell lysates of interphase and mitotic HeLa cells (Heusel et al., 2020) (Fig. S2C). We focused on the 54 proteins reported in Table 1. Interestingly, although most of the proteins that increased in mitotic blebs also increased in cells, for most proteins, the extent of the increase was higher in blebs than in cells. Furthermore, most proteins that showed decreased levels in mitotic blebs, displayed increased levels in mitotic cells. Together, these observations suggest that differences in cortical composition between interphase and mitosis are not only the result of differences in expression levels, but are tightly regulated through differential cortical recruitment.

Finally, we further scrutinised our dataset by comparing our list of 238 actin-related cortical proteins to a published targeted screen of proteins involved in the generation of the mitotic rounding force (Toyoda et al., 2017). We found that 103 out of the 238 actin-related proteins detected in blebs had been tested in this mechanical screen. Out of these, 10 were shown to reproducibly and significantly reduce mitotic rounding force upon depletion (Table S3). These 10 regulators of cortex mechanics included the heavy chain of non-muscle myosin IIA (MYH9), which served as a positive control in the mechanical screen (Toyoda et al., 2017), upstream regulators of actomyosin dynamics (RAC1 and ROCK2), the septin SEPT9, and proteins involved in the control of actin organisation. This last category included proteins affecting actin polymerisation and nucleation (DIAPH1, PFN1, DBN1 and CYFIP1), and actin bundling and crosslinking (ACTN4 and FSCN1). Mechanisms of how actin length regulators (e.g. DIAPH1 and PFN1) and crosslinkers (e.g. ACTN4) affect force generation in actomyosin networks have been explored previously (Logue et al., 2015; Ennomani et al., 2016; Chugh et al., 2017). Furthermore, MYH9 and SEPT9 were the only two proteins out of the 10 cortex mechanics regulators to also show a significant change in cortical levels in our mass spectrometry analysis (Table 1). We thus decided to focus on the role of septins, and septin 9 in particular, in cortex regulation for the rest of the study.

### A role for the septin protein family in the regulation of the mitotic cortex

**Fig. 4.**
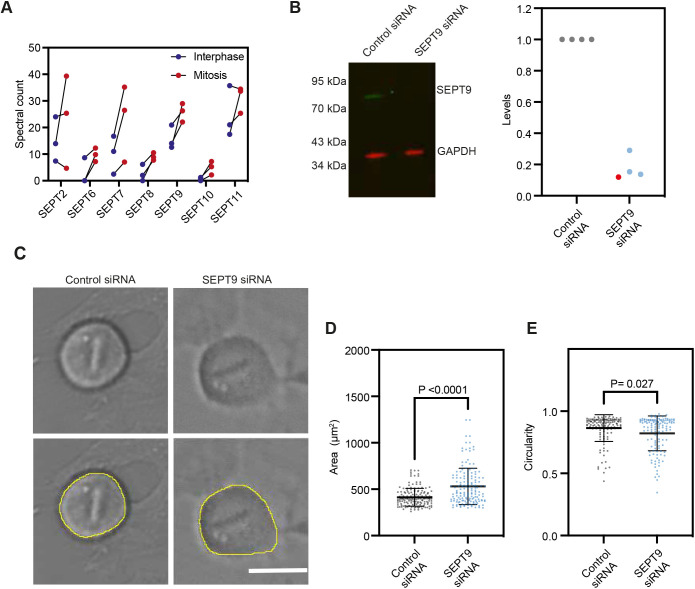
**Septin 9 regulates mitotic rounding.** (A) Normalised spectral counts for septins detected in the mass spectrometry analysis of interphase and mitotic blebs; each data point corresponds to an individual replicate. Unpaired one-tailed Student's *t*-test: *P*-values= 0.26, 0.050, 0.12, 0.017, 0.019, 0.024, 0.18 for SEPT2, 6, 7, 8, 9, 10 and 11, respectively. (B) Representative fluorescence western blot (left) and quantification (right) showing the decrease in septin 9 levels upon siRNA treatment. Membrane is representative of *n*=4 samples used for quantification. For quantification, protein levels were normalised to the loading control (GAPDH) and control siRNA conditions. Red datapoint on graph corresponds to the sample on the western blot membrane image (left panel). (C) Brightfield images of the cellular midplane of live mitotic cells treated with control and SEPT9 siRNA and example of manual segmentation (lower panel), that were used to analyse cell shape parameters. (D,E) Quantification of cell area (D) and circularity (defined as 4π*area/perimeter^2^, E) in mitosis of control and SEPT9 siRNA-treated cells. Scale bar: 20 µm. Graph, mean±s.d., three independent experiments, *n*=156 (area), *n*=126 (circularity) cells for each control and SEPT9 siRNA-treated cells. Statistics, Mann–Whitney test.

We next asked whether septins could play a role in the regulation of the cortex reorganisation between interphase and mitosis. Septins 2 and 9 have been previously shown to localise to the prometaphase cell cortex (Estey et al., 2010), but their role at the mitotic cortex remains unclear. Interestingly, our data indicate that the levels of several members of the septin family increase at the cortex between interphase and mitosis, with septin 8 and 9 displaying the most significant increase (Figs 3A and 4A). Proteins of the septin family assemble into higher-order structures, including filaments, and are increasingly considered a component of the cytoskeleton (reviewed in Mostowy and Cossart, 2012; Spiliotis, 2018). Septins are generally classified into four different homology subgroups (SEPT2, SEPT3, SEPT6 and SEPT7), with members of the same subgroup displaying some redundancy for the formation of oligomers and filaments. Septin 9 is the only member of the SEPT3 subgroup detected in blebs, and it also displayed a statistically significant change in cortical levels between interphase and mitosis in our analysis (Figs 3A and 4A, Table 1) and its depletion has been shown to significantly decrease mitotic rounding forces in a mechanical screen (Toyoda et al., 2017). We thus decided to first investigate the role of septin 9 in the regulation of the mitotic cortex.

We first assessed whole-cell levels of septin 9 and found that not only cortical (Fig. 4A) but overall septin 9 levels increase between interphase and mitosis (Fig. S3A), suggesting that septin 9 might play an important role in cell division. We thus depleted septin 9 (Fig. 4B,C) and examined the resulting effects on the cortex-driven mitotic rounding by analysing cell shape and cell division dynamics using live-cell imaging (Fig. 4C–E; Fig. S3B–D, Movies 1, 2). Septin 9-depleted cells showed strong mitotic rounding defects affecting metaphase cell shape and rounding time, whereas the cleavage ingression time was not significantly affected (Fig. 4D,E, Fig. S3C,D). In particular, septin 9-depleted cells took slightly longer to round up at the onset of mitosis (Fig. S3D) and displayed larger and less circular equatorial shapes in metaphase (as imaged in the cellular midplane, Fig. 4C–E), as well as a significantly higher aspect ratio (Fig. S3C) compared to control cells. Taken together, our dataset identifies septin 9 as an important regulator of cell rounding at the onset of mitosis.

Finally, we asked whether members of other septin homology groups also affected mitotic shape (Fig. S4). We tested how depletion of other septins affected metaphase cell circularity, as well as rounding and ingression times. We focused on septin 2 and septin 7, the only representatives of the SEPT2 and SEPT7 septin homology groups detected in isolated blebs (Fig. 4A), and septin 8, the member of the SEPT6 homology group that displayed the most significant change in levels between interphase and mitotic blebs (Fig. 4A; the other members of the SEPT6 group detected in blebs were septin 6, septin 10 and septin 11). We found that depletion of septin 2, septin 7 and septin 8 resulted in decreased equatorial circularity in metaphase cells, but we observed no significant change in mitotic rounding or ingression times with the exception of a slight increase in ingression time upon septin 7 depletion (Fig. S4B–D). Finally, to exclude potential compensation effects between different septins, we tested, using quantitative (q)PCR, how depletion of specific septins affected expression levels of other septins (Fig. S4A). Even though, following depletion of specific septins, slight changes in levels of some of the other septins were detected, these changes were small and did not suggest any clear pattern of compensation. Taken together, our data suggest that multiple members of the septin protein family act together to affect cell shape mechanics in early mitosis, highlighting the effectiveness of our dataset for identification of cell surface mechanics regulators.

## DISCUSSION

Here, we compared the composition of the actin cortex between interphase and mitosis, through mass spectrometry of cellular blebs isolated from synchronised rounded interphase and mitotic cells. Our previous work has shown that our purification protocol yields blebs displaying a cortex similar to the cellular cortex, as assessed by confocal and scanning electron microscopy, and that the isolated blebs display active contractions, confirming the presence of contractility regulators (Biro et al., 2013; Bovellan et al., 2014). We have verified that blebs isolated from cells synchronised in interphase and mitosis re-assemble an actin cortex (Fig. 1E; Fig. S1A), and are enriched in cortical components and depleted in nuclear components (Fig. S1B). Directly measuring cortical tension in isolated blebs is technically challenging due to their small size. Nonetheless, we observed that the blebs isolated from interphase cells tended to display less round shapes than those isolated from mitotic cells (see Fig. 1E for an example), suggesting that interphase blebs might display a lower cortical tension. Taken together, our observations indicate that bleb isolation constitutes a simple protocol for the purification of cellular fractions enriched for actin cortex components from interphase (low cortical tension) and mitotic (high cortical tension) cells. As a result, unlike previous studies where the protein composition of whole-cell lysates (Heusel et al., 2020) or re-polymerised F-actin-binding fractions (Serres et al., 2020) were compared between interphase and mitosis, our approach directly compares cortex-enriched cellular fractions. As such, our study can detect differences in protein levels at the cortex that are due to changes in either protein expression or protein localisation (Fig. S2C).

Our study identifies a dataset of 922 proteins detected in both interphase and mitotic blebs, out of which 238 are actin related. These 238 proteins represent a candidate list for the regulation of the cortex remodelling upon mitosis entry (Table S3). Analysis of our candidate list pointed to the septin family as a potential and underexplored regulator of the mitotic cortex. Septins were first identified as key regulators of yeast cytokinesis (reviewed in Gladfelter et al., 2001). In mammalian cells, several septins have also been shown to interact with actomyosin networks and with the plasma membrane (reviewed in Spiliotis and Nakos, 2021). Depletion of septins 2, 7 and 11 has been shown to affect cleavage furrow ingression, leading to multinucleated cells (Estey et al., 2010). In particular, septin 2 colocalises with non-muscle myosin II in dividing cells, potentially acting as a scaffold to promote myosin phosphorylation during cytokinesis (Joo et al., 2007). Furthermore, septin 9 depletion has been shown to interfere with midbody abscission following cytokinesis but did not affect cytokinesis (Estey et al., 2010; Kim et al., 2011). Interestingly, although septin 9 and septin 2 localise to the mitotic cortex during prometaphase (Estey et al., 2010), whether septins also affect the cortex and cellular shape during earlier stages of mammalian cell division, prior to cytokinesis, had previously received little attention. Our study suggests that septins already regulate the cortex in the very early stages of mitosis, and contribute to the changes in cell surface mechanics that drive cell rounding. We showed that members of all four septin homology groups affect metaphase cell shape. Septins from different groups are required for the formation of septin oligomers and filaments (reviewed in Mostowy and Cossart, 2012). How exactly specific septins are recruited to the mitotic cortex, and whether they regulate mitotic cortex mechanics and resulting cell rounding independently or act together through the formation of multimeric complexes and higher order structures, will constitute interesting avenues for future studies.

The identification of septins as regulators of the cortex-driven cell shape changes at mitosis entry highlights the potential of the dataset we generated in detecting important cortex regulators among the multiple cortical components. More broadly, by systematically comparing the composition of the mitotic and interphase actin cortex, our study will be an important resource for investigations of mitotic shape changes, and of actin cortex mechanics in general.

## MATERIALS AND METHODS

### Cell culture, cell synchronization and siRNA treatment

HeLa cells from MPI-CBG Technology Development Studio (TDS) were cultured in DMEM Glutamax (Thermo Fisher Scientific) supplemented with 10% fetal bovine serum (FBS; Thermo Fisher Scientific), 1% penicillin-streptomycin (Thermo Fisher Scientific) and 1% L-glutamine (Thermo Fisher Scientific). For passaging, cells were detached from culturing flasks with Trypsin-EDTA (Thermo Fisher Scientific). Cells were regularly tested for mycoplasma.

Cells were synchronised in interphase with 2 mM thymidine for ∼22 h, and in prometaphase with 2 μM S-trityl-L-cystine (STLC) for ∼16 h. Mitotic cells were further enriched by undertaking a mitotic shake-off. Thymidine and STLC were removed from the cells before bleb isolation.

For siRNA-mediated depletion, cells were transfected with ON-TARGET plus Human SEPT9 pool siRNA (Horizon Discoveries, 006373-00), ON-TARGET plus Human SEPT2 pool siRNA (Horizon Discoveries, 010614-00), ON-TARGET plus Human SEPT7 pool siRNA (Horizon Discoveries, 011607-00), ON-TARGET plus Human SEPT8 pool siRNA (Horizon Discoveries, 010647-00) or non-targeting control pool siRNA (Horizon Discoveries, 001810-10) using Lipofectamine™ RNAiMAX Transfection Reagent (Thermo Fisher Scientific) in antibiotic-free medium. Overnight imaging was performed 24–48 h after siRNA treatment and SEPT9 levels were checked by western blotting 48 h after the treatment.

### Bleb isolation

Blebs were isolated from either mitotic cells synchronised with STLC and detached by undertaking a mitotic shake-off, or from cells synchronised in interphase with thymidine and detached with trypsin (at 37°C). Trypsin was deactivated through a dilution with cell culturing medium, followed by centrifugation at 164 ***g*** for 3 min and exchanging medium for fresh culturing medium. Blebbing was induced by addition of either 1.7 μM (interphase) or 2.4 μM (prometaphase) of latrunculin B (Sigma Aldrich), immediately followed by shaking on a horizontal benchtop shaker for 15 min at room temperature to detach the blebs from the cells. Latrunculin was then washed out through centrifugation of isolated blebs (and cells) at 4410 ***g*** for 6 min and re-suspension in intracellular buffer (0.01 M sodium chloride, 0.28 M pH 7.2 L-glutamic acid, 0.014 M magnesium sulphate, 0.013 M calcium chloride, 0.020 M pH 6.8 EGTA, 0.04 M pH 7.2 HEPES, in distilled H_2_O; potassium hydroxide to adjust the pH of L-glutamic acid, EGTA and HEPES). To separate entire cells and isolated blebs, cells were firstly pelleted with a 4 min centrifugation at 100 ***g***. The supernatant was then filtered with 5 μm Satorious Minisart filters (FIL6602, Minisart) to remove remaining cells and any larger debris. Collected blebs were then pelleted with a centrifugation at 16,100 ***g*** for 5 min and incubated in a solution containing an exogeneous ATP regeneration system (energy mix) and α-toxin to permeabilise the bleb membrane [5% A-haemolysin α-toxin (1 mg ml^−1^; H9395, Sigma-Aldrich), 2% energy mix (50 mg ml^−1^ UTP, 50 mg ml^−1^ ATP, 255 mg ml^−1^ creatine phosphate), 2% creatine kinase (10 mg ml^−1^), in intracellular buffer] for 10 min, followed by centrifugation at 16,100 ***g*** for 5 min to remove α-toxin and resuspension of blebs in 500 μl re-suspension buffer (50% intracellular buffer, 44% distilled H_2_O, 1% energy mix, 5% creatine kinase) for 20 min. Bleb isolation was performed at room temperature. Purified blebs were then lysed directly in the Laemmli sample buffer and the lysates were prepared for mass spectrometry.

### Mass spectrometry

To obtain sufficient material for MS analysis, isolated blebs were prepared from 15 T175 flasks containing cells synchronised in interphase and 60 T175 flasks of cells synchronized in mitosis, in three experimental replicates for each phase of the cell cycle. After bleb isolation, samples were subjected to SDS-PAGE and Coomassie-stained gel bands were excised and subjected to in-gel trypsin digestion, as described previously (Carrière et al., 2008). The resulting peptides were extracted and subjected to capillary LC-MS/MS using a high-resolution hybrid mass spectrometer LTQ-orbitrap XL (Thermo Fisher Scientific). Experiments were performed in triplicates. Database searches were performed against Uniprot SwissProt Human database (containing 20,347 protein entries) using PEAK Studio (version 8.5) as search engine, with trypsin specificity and three missed cleavage sites allowed. Methionine oxidation, lysine acetylation, cysteine carbamidomethylation, serine/threonine/tyrosine phosphorylation and asparagine/glutamine deamidation were set as variable modifications. The fragment mass tolerance was 0.01 Da and the mass window for the precursor was ±10 ppm. The data were visualised with Scaffold (version 4.8.6) and minimum number of peptides per protein was set to two for data analysis. For normalisation, the total number of spectra identified for each protein was divided by the total spectral count detected in the specific replicate considered, and multiplied by the total spectral count detected in the first interphase replicate, thus normalising for experimental variation between replicates.

### Gene Ontology and candidate list curation

For GO analysis, the statistical overrepresentation test function from PantherDB (http://www.pantherdb.org/) was used. Actin-related proteins from the bleb extracts were identified by comparing the list with previous mass spectrometry analysis of the F-actin interactome in interphase and mitotic cells (Serres et al., 2020), and by manually adding known actin related proteins.

### Western blotting

Cells were lysed directly in Laemmli sample buffer, boiled and sonicated. 30 µg of total protein per sample was loaded on NuPage 4-12% Bis-Tris Protein gels (Thermo Fisher Scientific) or 4–15% Mini-PROTEAN TGX Stain-Free Protein gels (Bio-Rad) and run at 200 V as per the manufacturer's instructions. Proteins were transferred onto a nitrocellulose membrane (Thermo Fisher Scientific) using the Bio-Rad transfer system at 100 V for 60 min at 4°C or with Trans-Blot Turbo Mini PVDF Transfer Packs using Trans-Blot Turbo Transfer System (Bio-Rad). The membrane was blocked for 30 min with the Odyssey Blocking Buffer (TBS) (Licor) and stained overnight with primary antibodies at 4°C. Primary antibodies used were against: phospho-histone H3 (Cell Signalling, 9713S, 1:500 dilution), β-actin (Santa Cruz Biotechnology, 47778), cyclin B (Santa Cruz Biotechnology, 245) septin 9 (Sigma-Aldrich, HPA042564), GAPDH (Abcam, 8245, 1:5000 dilution), filamin A (Santa Cruz Biotechnology, 28284), ezrin-radixin-moesin (Cell Signalling, 3142), myosin RLC (Cell Signalling, 3672), phospho-myosin RLC (Cell Signalling, 3675), Histone H3 (Abcam, 1791) in 5% milk in PBS with 0.1% Tween 20. Antibodies were diluted 1:1000 unless stated otherwise. Following primary antibody incubation, membranes were washed three times with PBS with 0.1% Tween 20. Licor secondary antibodies conjugated to IRDyes were diluted 1:5000 in 5% milk in PBS with 0.1% Tween 20 and incubated with the membranes at room temperature for 60 min. Membranes were imaged with Odyssey FC system and the results were analysed with the Studio Lite software. Uncropped images of western blots from this paper are shown in Fig. S5.

### qPCR

Cells were collected 24 and 48 h after siRNA treatment, and total RNA was extracted by using an RNeasy mini Kit (Qiagen) and reverse-transcribed using a cDNA Reverse Transcription Kit (Applied Biosystems), as described by the manufacturer. For each qPCR assay, a standard curve was performed to ensure the efficacy of the assay (between 90 and 110%). The Viia7 qPCR instrument (Life Technologies) was used to detect amplification level and was programmed with an initial step of 20 s at 95°C, followed by 40 cycles of 1 s at 95°C and 20 s at 60°C. Relative expression (RQ=2^−ΔΔCT^) was calculated using the Expression Suite software (Life Technologies), and normalization was done using both ACTB and GAPDH.

Levels of septins were measured using the following primers: SEPT2 forward, 5′-AAGGCAATACACAACAAGGTGA-3′, reverse- 5′-TTCTTCAATTTCATCCAGAATCC-3′; SEPT6 forward, 5′-TCGTCCAGCGAGTCAAAGAG-3′, reverse, 5′-TCTCGTCCTGGTGCAGTTTC-3′; SEPT7 forward, 5′-GAAGTTAATGGCAAAAGGGTCA, reverse, 5′-TCAAGTCCTGCATGTGTGTTC-3′; SEPT8 forward, 5′-TTCAGGACAGCGATGGTGAC, reverse, 5′-CTCCTTCCTCTGCAGCTCAC-3′; SEPT9 forward, 5′-CGGGACCTTCTCATCAGG, reverse, 5′-GGTACGCCTCGAAGTGGAT; SEPT10 forward, 5′-GTTGCTTCTGCCCTCCGG, reverse, 5′-CGGTGAAGCGGCTGTATCAG-3′; SEPT11 forward, 5′-TTACTACAGTCCCAGGCCCA, reverse, 5′-TGGCTTGCCAGGCTTTATGT-3′. ACTB and GAPDH were used as endogenous controls using the following primers ACTB forward, 5′-CCATCTACGAGGGGTATGCC-3′, reverse, 5′-GCGCTCGGTGAGGATCTTC-3′; GAPDH forward, 5′-AGCCACATCGCTCAGACAC-3′, reverse, 5′-GCCCAATACGACCAAATCC-3′.

### Preparation of fixed samples for imaging

Samples, isolated blebs and cells were spun on poly-L-lysine coated 25 mm coverslips by centrifugation at 460 ***g*** for 10 min. Cells were fixed with 4% paraformaldehyde (PFA) in PBS for 10 min followed by 10 min permeabilisation with 0.2% Triton X-100 at room temperature. Blebs were fixed with combined permeabilisation-fixation for 6 min with 4% PFA in intracellular buffer with 0.2% Triton X-100 followed by 14 min fixation with 4% PFA in intracellular buffer at room temperature, followed by three washes with PBS. Samples were stained with DAPI and phalloidin–Alexa568 (1:500 dilution, Thermo Fisher Scientific, A12380) for 1 h, followed by three washes with PBS.

Samples were imaged using Olympus FluoView FV1200 Confocal Laser Scanning Microscope using a 60× oil objective (NA 1.4).

### Live imaging and analysis

Cells we treated with siRNA for 24 h before the start of the overnight imaging. 30 min prior to imaging, the culture media was changed to Leibovitz's L-15 (Thermo Fisher Scientific) media supplemented with 10% FBS and 1% Penicillin-Streptomycin. Cells were imaged for 15 h at 37°C every 2 min on an inverted Olympus IX81 microscope, controlled via Velocity interface, and a Hamamatsu Flash 4.0v2 ScMOS camera. Imaging was performed with the brightfield setting under a 20× air objective.

Cell shape was analysed with manual segmentation using the measure feature of the FIJI image analysis software (Schindelin et al., 2012). Mitotic cell shape was measured in the cellular midplane (Fig. 4C; Fig. S3B), at the last frame of metaphase, i.e. the last frame where aligned chromosomes were observed, prior to anaphase onset and chromosome segregation. For a small subset of cells, even after cell rounding, the cells maintained protrusions that extended from the cell surface (see example in Fig. S3B). These protrusions were included in the cell contour; they occurred with comparable frequency in control and SEPT9 siRNA-treated cells. Rounding time was measured from the first frame where retraction of cell edges was observed (onset of rounding) to the first frame of completely round morphology; in cases where active rounding continued throughout metaphase, the end of rounding was set at the end of metaphase (last frame before chromosome separation onset). Ingression time was measured from the last frame where aligned chromosomes were observed (just before the onset of chromosome segregation) until the end of furrow ingression. Statistical analysis was performed for individual cells pooled over three independent experiments, sample numbers are reported in the figure legends.

### STORM sample preparation, imaging and analysis

Samples were spun onto poly-L-lysine-coated 35 mm Dish High Precision 1.5 Coverslips (MatTek, P35G-0.170-14-C) by centrifugation at 460 ***g*** for 10 min. Samples were fixed for 6 min with 4% PFA in intracellular buffer with 0.2% Triton X-100 followed by 14 min with 4% PFA in intracellular buffer and further permeabilised for 10 min with 0.5% Triton X-100 in PBS. Samples were blocked for 30 min in 5% bovine serum albumin (BSA) in PBS before staining. Samples were stained by 1 h incubation with 1:200 phalloidin–Alexa-Fluor-647 (Thermo Fisher Scientific, A22287) diluted in PBS.

Samples were imaged with a buffer of 45 mM Tris-HCl, 10 mM NaCl, 10% glucose, 5 U/ml pyranose oxidase, 35 mM MEA, 40 µg/ml catalase and 2 mM cyclooctatetraene (COT) as previously described (Olivier et al., 2013).

Samples were imaged using the Zeiss Elyra 7 imaging set-up for single-molecule localisation using 64× objective with NA 1.46. Laser power was at 100% with 20 ms exposure for 10,000 frames. Acquisitions were corrected for drift using model-based correction, grouped and filtered for localisation precision 5–50 nm and number of photons 500–5000 using Gaussian distribution with the in-built Zeiss Black processing software.

### Statistical analysis

Mass spectrometry results (Table 1; Tables S1–S3) were analysed using Excel Microsoft and Prism (GraphPad Software) was used for analysis of all other experiments. To compare means unpaired one-tailed Student's *t*-test was used or Mann–Whitney test for non-normally distributed samples as specified in the figure legends.

## Supplementary Material

Supplementary information

Reviewer comments
