# Supplementary material for: Proteomic analysis of the actin cortex in interphase and mitosis
Source: J Cell Sci. 2022 Aug 26;135(16):jcs259993. doi: 10.1242/jcs.259993 (PMC9481927; doi:10.1242/jcs.259993)
Supplement: Supplementary information [file joces-135-259993-s1.pdf]

Figure S1

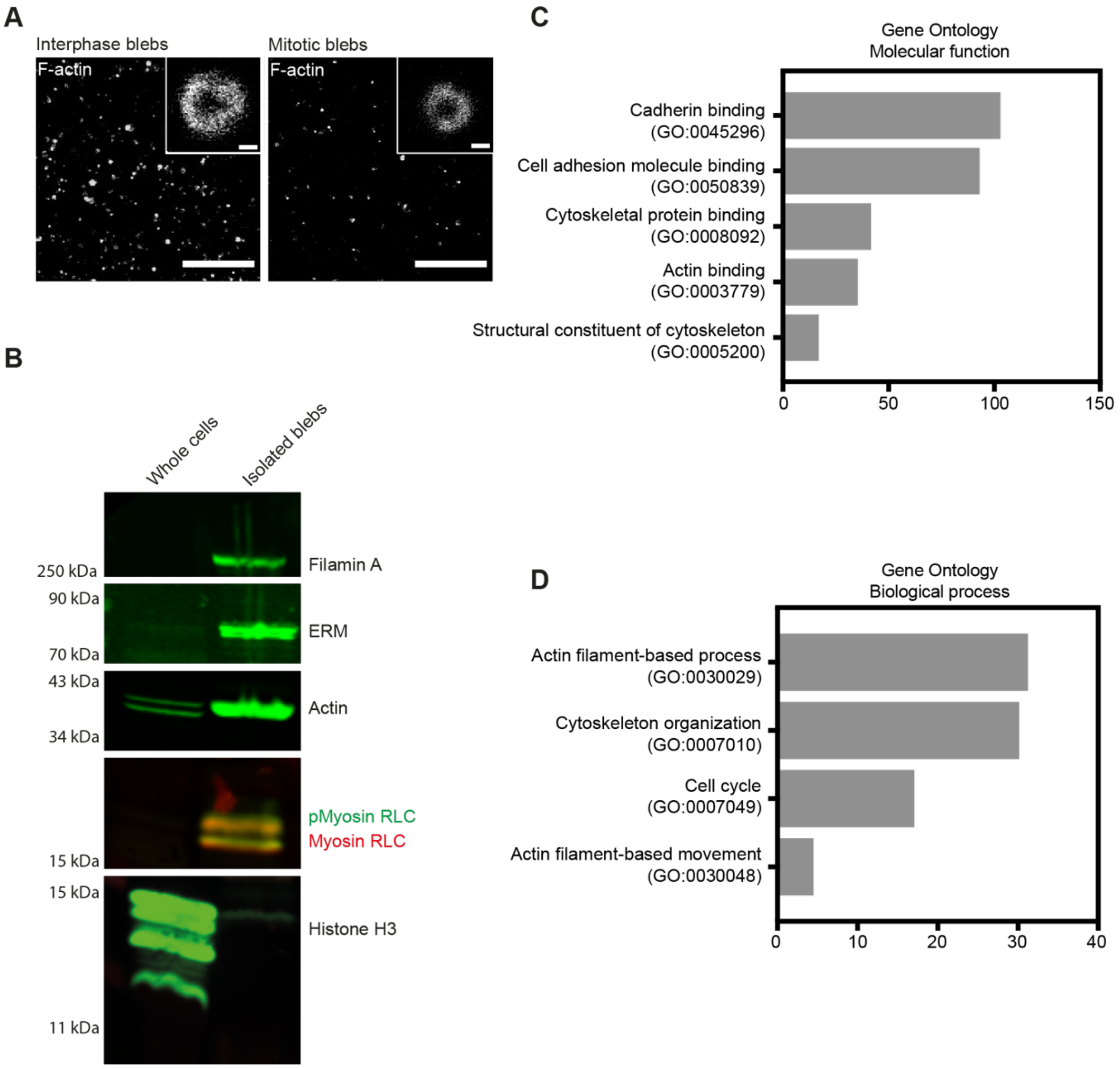

Fig. S1. Analysis of isolated blebs. (A) Representative confocal images of the actin cortex in blebs isolated from interphase (left) and mitotic (right) cells used for the proteomic analysis. The cortex was re-assembled after addition of an ATP regeneration system. White: phalloidin (F-actin). Insets: zoom-ed in individual blebs. Scale bars: 20 μm; 0.5 μm (inset). (B) Fluorescent western blot for filamin A, ERM (ezrin, radixin, moesin), actin, myosin regulatory light chain (RLC), phospho myosin RLC, actin and nuclear protein (histone H3) in whole cells and in isolated blebs (from unsynchronised cells). Equal amounts of total protein were loaded for western blot analysis as measured with Pierce Protein Assay. (C, D) Gene Ontology (GO) analysis of the selected 922 proteins, focusing on GO terms for molecular function (C) and biological function (D) related to the cell cortex.

Figure S2

A

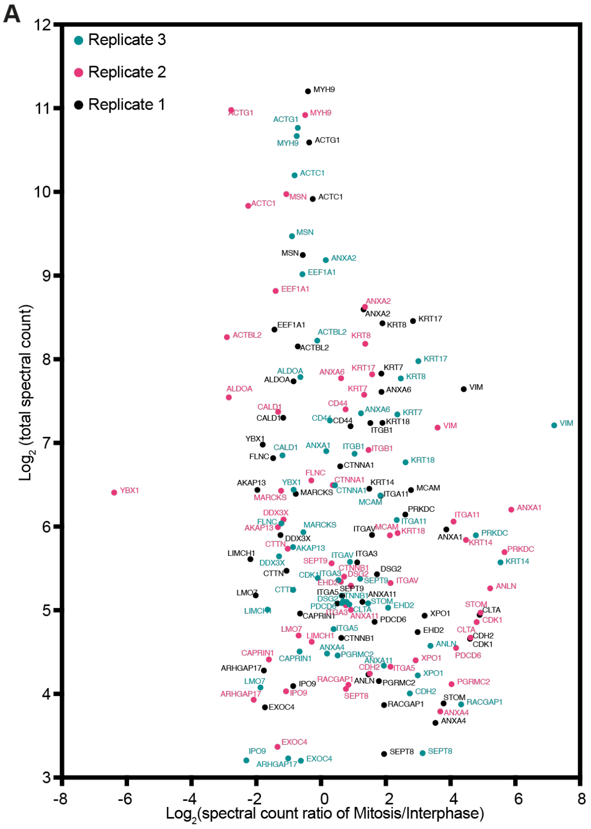

B

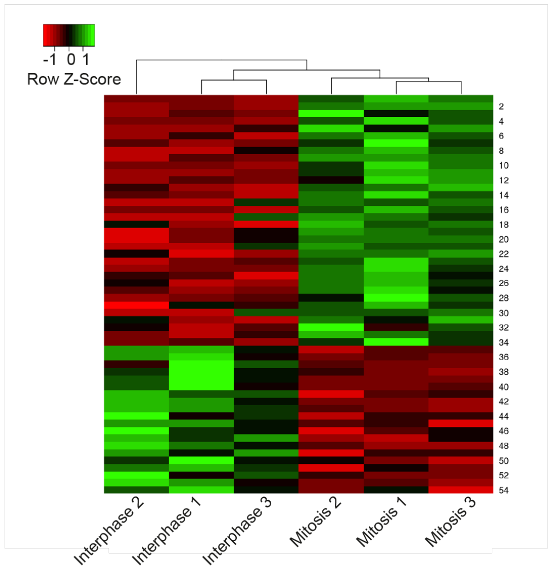

C

Mitotic decrease in blebs increase in cells			
Protein	Log_2FC bleb	Log_2FC cell	Ratio FC (bleb/cell)
FLNC	-0.971	0.0164	0.504
MYH9	-0.515	0.575	0.470
MSN	-0.889	0.447	0.396
CTTN	-1.00	0.365	0.388
ALDOA	-1.22	0.170	0.382
CALD1	-1.22	0.334	0.341
ACTBL2	-1.06	0.550	0.328
LIMCH1	-1.51	0.120	0.320
EXOC4	-1.29	0.370	0.317
IPO9	-1.22	0.487	0.307
ARHGAP17	-1.69	0.298	0.252
YBX1	-2.00	0.188	0.219
CAPRIN1	-0.916	1.80	0.152
LMO7	-1.47	5.05	0.0108

Mitotic decrease in blebs and cells			
Protein	Log_2FC bleb	Log_2FC cell	Ratio FC (bleb/cell)
MARCKS	-0.889	-1.76	1.83
AKAP13	-1.43	-0.179	0.419

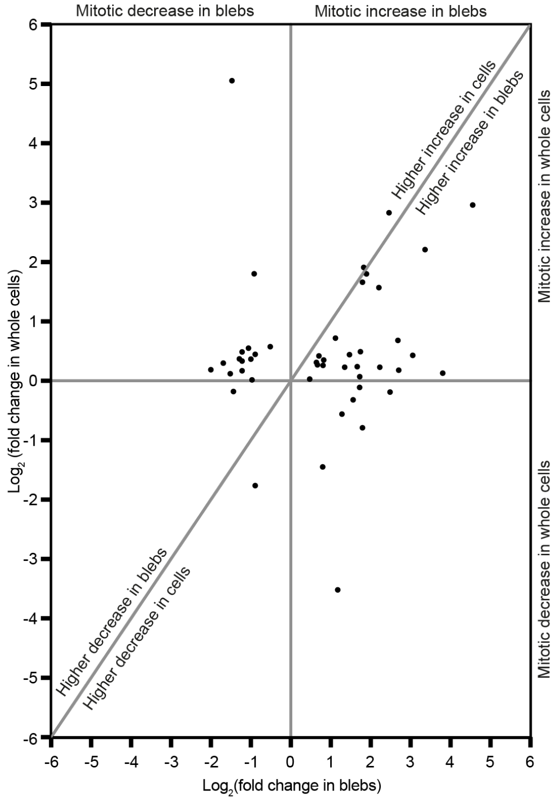

Mitotic increase in blebs and cells			
Protein	Log_2FC bleb	Log_2FC cell	Ratio FC (bleb/cell)
PRKDC	3.80	0.132	12.7
XPO1	3.05	0.427	6.18
CDH2	2.70	0.177	5.76
CLTA	2.68	0.682	4.00
MCAM	2.23	0.230	4.00
SEPT8	1.73	0.068	3.16
VIM	4.55	2.96	3.01
PDCD6	1.66	0.236	2.69
CDK1	1.74	0.494	2.38
ANLN	3.36	2.21	2.23
ITGB1	1.35	0.231	2.17
ITGAV	1.47	0.438	2.05
KRT18	2.20	1.57	1.55
ITGA5	0.807	0.258	1.46
ITGA3	0.824	0.346	1.39
CTNNA1	0.475	0.0265	1.37
DSG2	1.12	0.719	1.32
CTNNB1	0.669	0.270	1.32
CD44	0.642	0.307	1.26
SEPT9	0.705	0.416	1.22
KRT7	1.80	1.66	1.10
RACGAP1	1.90	1.80	1.07
KRT8	1.83	1.91	0.945
KRT17	2.46	2.83	0.774

Mitotic increase in blebs decrease in cells			
Protein	Log_2FC bleb	Log_2FC cell	Ratio FC (bleb/cell)
ANXA6	1.18	-3.52	26.0
ITGA11	2.49	-0.186	6.37
ANXA4	1.80	-0.787	5.60
ANXA2	0.800	-1.45	4.74
EHD2	1.56	-0.321	3.68
ANXA11	1.28	-0.556	3.57
PGRMC2	1.72	-0.11	2.75

Journal of Cell Science • Supplementary information

Fig. S2. Changes in levels of significantly changing actin binding proteins in blebs between interphase and mitosis. (A) Total interphase and mitosis spectral counts (log scale, y-axis) for the 54 actin-related proteins that significantly change in levels between interphase and mitotic blebs (listed in Table 1); x-axis shows enrichment between interphase and mitosis. Datapoints for all three experimental replicates are shown. (B) Heatmap of the levels (normalised spectral counts) of the 54 actin-related proteins listed in Table 1 in the different experimental replicates of interphase and mitotic blebs. (C) Mitotic enrichment of the 54 actin-related proteins reported in Table 1, in isolated blebs (x-axis) vs whole cells (y-axis). Protein levels for whole cells were taken from Heusel et al., 2020, where cells were synchronised in interphase using a double thymidine block and in mitosis with a thymidine block followed by synchronisation with nocodazole and mitotic shake-off. Mean spectral counts across 3 replicates of blebs were used to calculate enrichment. Spectral counts were normalised to account for variation between experiments.

Figure S3

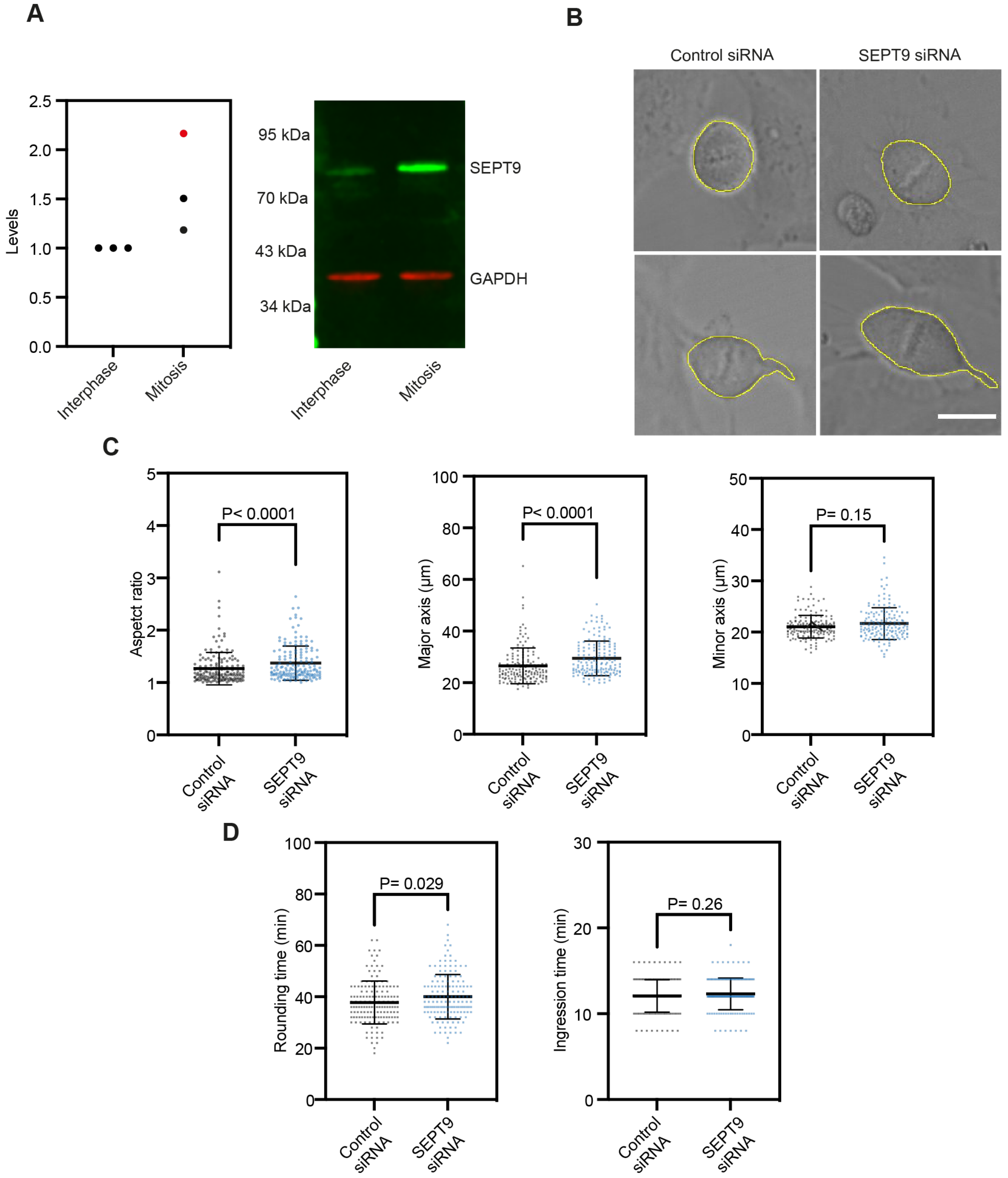

Fig. S3. Role of septin 9 in mitosis. (A) Representative fluorescent western blot (left panel), and related quantification (right panel), of septin 9 levels in mitotic and interphase whole cell lysates, normalised to the loading control (GAPDH) and relative to interphase levels. Membrane is representative of n=3 samples used for quantification. Red datapoint on the graph corresponds to the sample on the western blot membrane image (left panel). (B) Example of brightfield images of the cellular midplane of live mitotic cells treated with control and SEPT9 siRNA, that were used to analyse cell shape parameters (panel C and also Fig. 4D,E). A small subset of the cells, both in controls and in SEPT9 siRNA treated cells, displayed large protrusions

Journal of Cell Science • Supplementary information

that did not retract during cell rounding (example pictures in lower row); these protrusions were included in cell shape quantification. Scale bar: 20 μm. (C) Quantification of cellular aspect ratio, major axis, and minor axis in mitosis in control and SEPT9 siRNA treated cells, n=156 for each control and SEPT9 siRNA treated sample. (D) Rounding and furrow ingression times in control and septin 9-depleted cells, n=149 (SEPT9 siRNA) and 151 (control). Graph, mean ± 1 standard deviation, 3 independent experiments; Statistics: Mann-Whitney test.

Figure S4

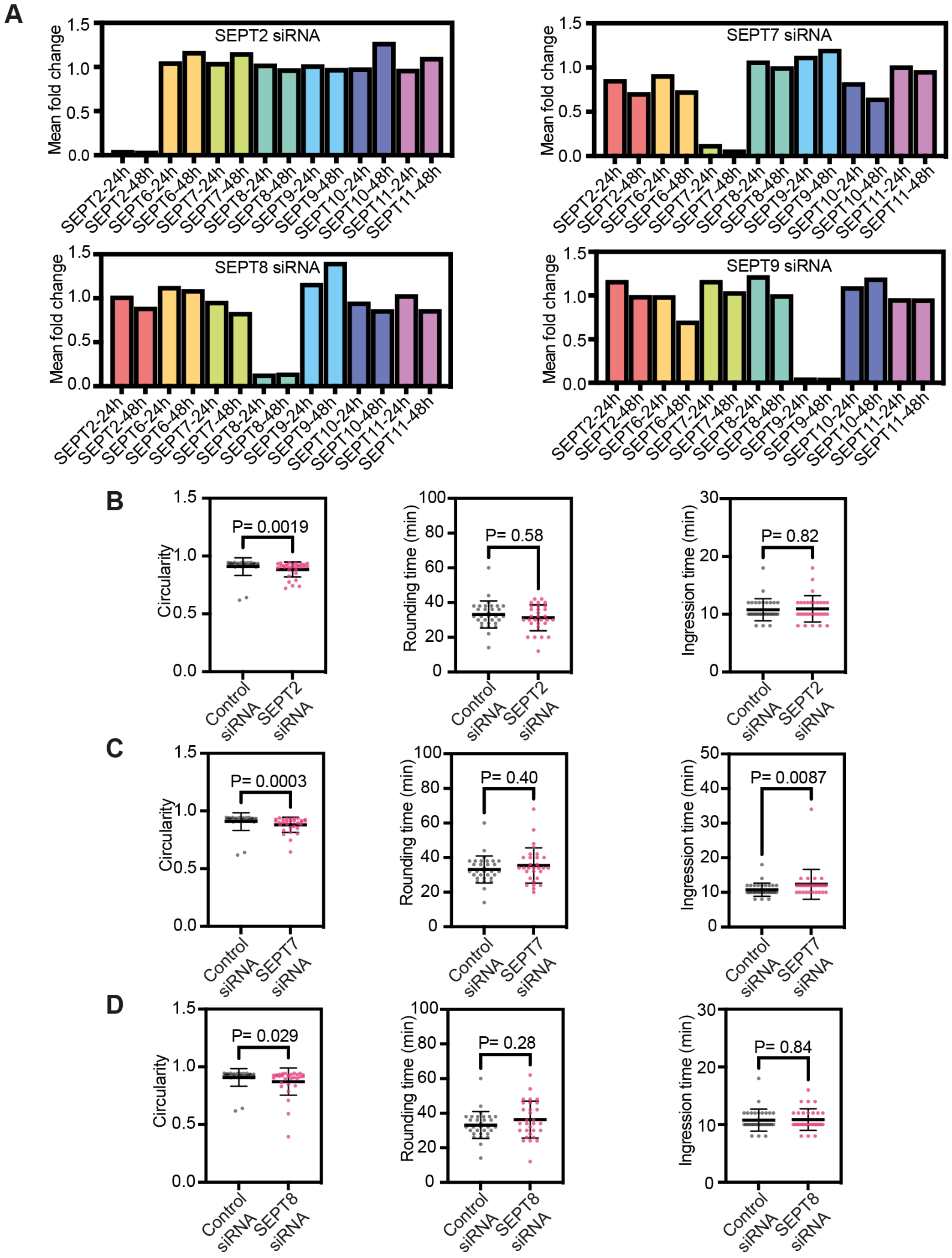

Fig. S4. Role of other septins in mitosis. (A) Cellular expression levels of SEPT2, SEPT6, SEPT7, SEPT8, SEPT9, SEPT10, SEPT11 24h and 48h after treatment with SEPT2, SEPT7, SEPT8, or SEPT9 siRNA. Levels are displayed as fold change compared to the levels observed in samples treated with control siRNA. Mean change of three experiments is displayed. (B-D) Quantification of circularity of metaphase cells, rounding and furrow ingression time for cells treated with control and (B) SEPT2, (C) SEPT7, (D) SEPT8 siRNA. Graph, mean ± 1 standard deviation, 3 independent experiments, n=30 for each sample, Statistics: Mann-Whitney test.

Journal of Cell Science • Supplementary information

Blot Transparency.

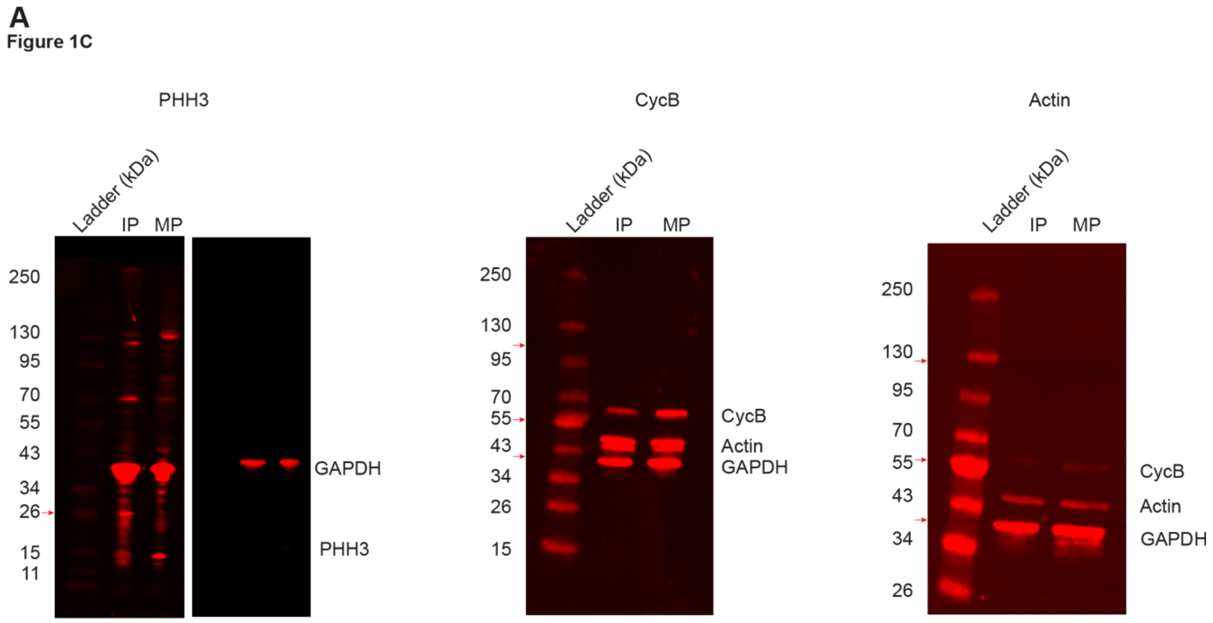

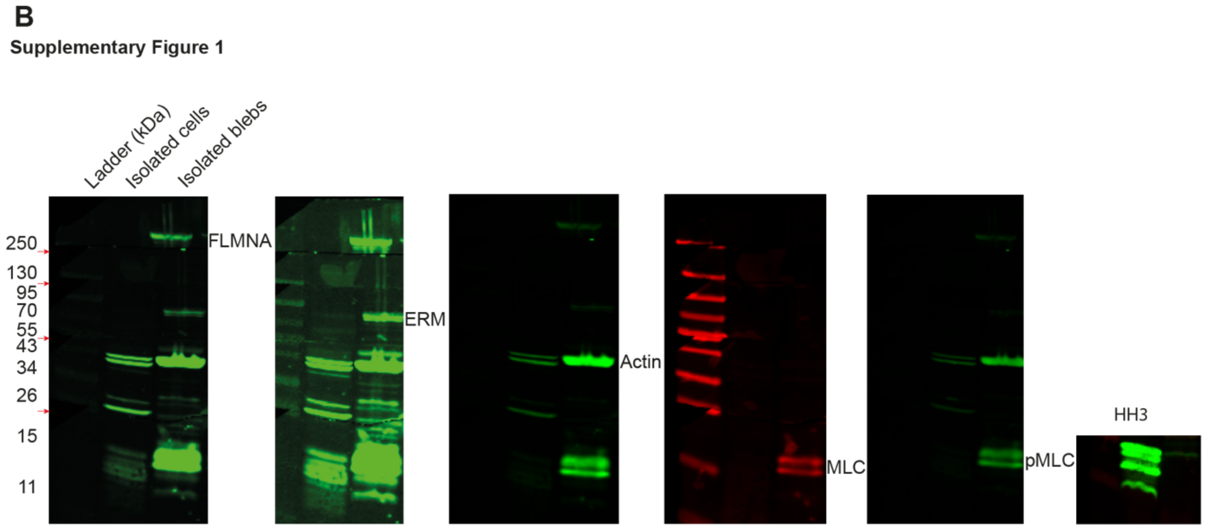

Fig. S5. Blot Transparency. Uncropped western blots corresponding to the blots displayed in various figures.

(A) Uncropped western blots for Figure 1C. PHH3: Uncropped blot stained for PHH3 and GAPDH. Left: Exposure used for imaging and quantification of PHH3, in which GAPDH signal was overexposed. Right: Exposure used for imaging of GAPDH signal. The membrane was cut at ~26 kDa, which can be noticed through misaligned protein markers at the corresponding molecular weight. CycB: Uncropped blot stained for CycB, actin and GAPDH. This image was used in Figure 1C as an example of cyclin staining. The membrane was cut at at ~40 kDa, ~55 kDa, and ~100 kDa, which can be noticed through misaligned protein markers at the corresponding molecular weights. Actin: Uncropped blot stained for CycB, actin and GAPDH. This image was used in Figure 1C as an example of actin staining. The membrane was cut at at ~40 kDa, ~55 kDa, and ~100 kDa, which can be noticed through misaligned protein marker

Journal of Cell Science • Supplementary information

at the corresponding molecular weights. Protein Ladder: PageRuler Prestained NIR Protein Ladder. (B) Uncropped western blots for Figure S1. Images of the same blot at different exposures, adjusted for quantification of levels of specific proteins highlighted on the right-hand side of each membrane image. The membrane was cut at the triangular marks noticeable on the left side of the blot. Before staining for HH3, the membrane (between 11 and 15 kDa) was stripped. Protein Ladder: PageRuler Prestained NIR Protein Ladder.

Red arrows indicate where the membranes were cut for antibody incubation and re-aligned for imaging.

Uncropped blots are not provided for Figure 4D and Supplementary Figure 4B as these figures already display large sections of uncropped blots.

Table S1. Proteins detected in blebs. Normalised spectral counts of all proteins detected by mass spectrometry of isolated blebs.

Click here to download Table S1

Table S2. Proteins detected in both interphase and mitotic blebs. Mean (normalised) spectral counts in interphase and mitosis, ratio of the means between mitosis and interphase (calculated from 3 replicates), and P-value calculated with Student's t-test, for the 922 proteins detected in both interphase and mitosis isolated bleb samples. Manually selected actin-related proteins (see Main text for list curation criteria) are highlighted in yellow.

Click here to download Table S2

Table S3. **Actin-related proteins detected in blebs.** Average PAI (spectra count normalised to molecular weight), ratio of the means, and P-value (calculated with Student's t-test) for the 238 actin-related proteins detected with blebs. Right column: rounding force changes upon esiRNA treatment against the proteins (as reported in Toyoda et al., 2017). Not tested: protein not examined; No change: protein for which no change was detected with any of the esiRNA tested; Potentially lower force: protein for which the change in rounding force was detected with some of the esiRNA sequences tested; Lower force: protein for which rounding force changed with all esiRNA sequences tested in (Toyoda et al., 2017).

Click here to download Table S3

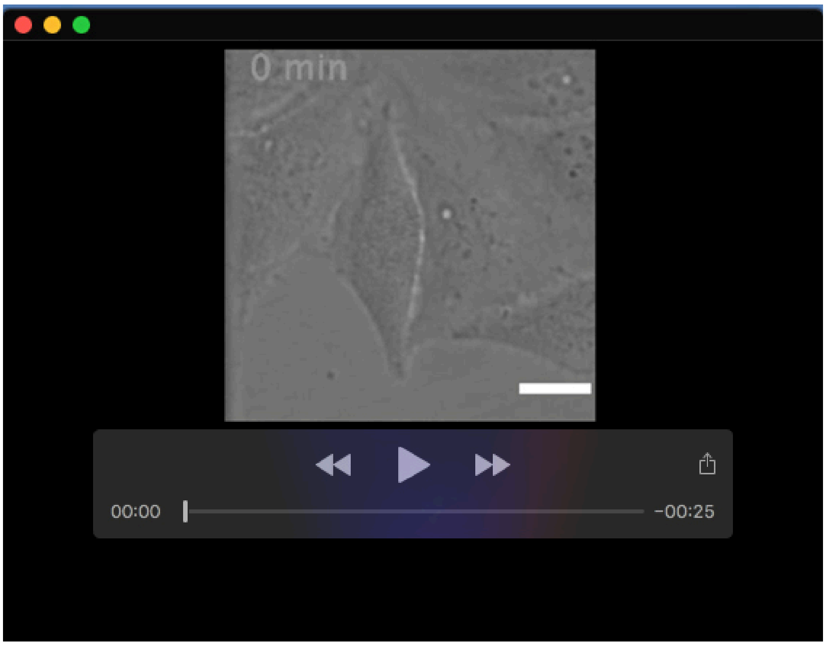

Movie 1. Dividing cell transfected with control siRNA. Movies from live-cell imaging were used for quantification of cell shape (Figure 4D-E, Supplementary Figure 4D). Time resolution= 2 min. Scale bar = 20 μm.

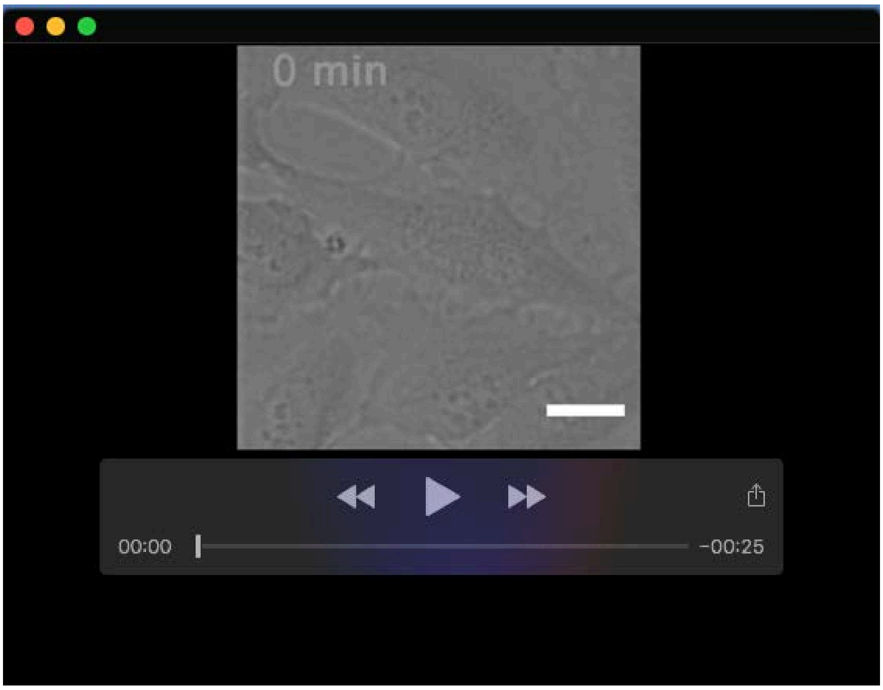

Movie 2. Dividing cell transfected with SEPT9 siRNA. Movies from live-cell imaging were used for quantification of cell shape (Figure 4D-E, Supplementary Figure 4D). Time resolution= 2 min. Scale bar = 20 μm.

Journal of Cell Science • Supplementary information
