# Supplementary material for: Proteomic analysis of the actin cortex in interphase and mitosis
Source: J Cell Sci. 2022 Aug 26;135(16):jcs259993. doi: 10.1242/jcs.259993 (PMC9481927; doi:10.1242/jcs.259993)
Supplement: Reviewer comments [file joces-135-259993_review_history.pdf]

Journal of Cell Science

Proteomic analysis of the actin cortex in interphase and mitosis

Neza Vadnjal, Sami Nourreddine, Geneviève Lavoie, Murielle Serres, Philippe P. Roux and Ewa K. Paluch
DOI: 10.1242/jcs.259993

Editor: Michael Way

Review timeline

Original submission:	4 March 2022
Editorial decision:	5 April 2022
First revision received:	6 July 2022
Accepted:	7 July 2022

Original submission

First decision letter

MS ID#: JOCES/2022/259993

MS TITLE: Proteomic analysis of the actin cortex in interphase and mitosis

AUTHORS: Neza Vadnjal, Sami Nourreddine, Genevieve Lavoie, Murielle Serres, Philippe P Roux, and Ewa K Paluch
ARTICLE TYPE: Tools and Resources

We have now reached a decision on the above manuscript.

To see the reviewers' reports and a copy of this decision letter, please go to: https://submit-jcs.biologists.organd click on the 'Manuscripts with Decisions' queue in the Author Area. (Corresponding author only has access to reviews.)

As you will see, the three reviewers raise a number of criticisms that prevent me from accepting the paper at this stage. They suggest, however, that a revised version might prove acceptable, if you can address their concerns. I am not expecting a complete septin story but it would be nice to have a bit more depth on the septin side of things, say for example the timing of mitosis and a knockdown of Septin 8 given the comments from reviewer 3.

Please ensure that you clearly highlight all changes made in the revised manuscript. Please avoid using 'Tracked changes' in Word files as these are lost in PDF conversion.

I should be grateful if you would also provide a point-by-point response detailing how you have dealt with the points raised by the reviewers in the 'Response to Reviewers' box. Please attend to all of the reviewers' comments. If you do not agree with any of their criticisms or suggestions please explain clearly why this is so.

Reviewer 1

Advance summary and potential significance to field

In Vadnjal, N et al., the proteomic composition of the cortical actin network in interphase and mitotic cells is resolved by mass spectrometry. To obtain a pure cortical actin network, which

© 2022. Published by The Company of Biologists under the terms of the Creative Commons Attribution License (https://creativecommons.org/licenses/by/4.0/).

includes numerous associated factors, the authors use a previously devised protocol for isolating blebs. By comparing the cortical actin network in interphase and mitotic cells, the authors identify specific factors required for mitotic cell rounding – a process requiring a heightened level of cortical actomyosin contractility. Thus, factors enriched in blebs from mitotic cells are likely to play a role in this process. Indeed the authors validate a candidate factor, septin 9, is required for mitotic cell rounding. Due to the involvement of the cortical actin network in numerous processes, this is likely to be a valuable resource to a wide array of cell biologists.

Comments for the author

Results:
1. Lines 71-74, as this phenomenon is important to the underlying premise of the article, it would be helpful to be more specific about how cortical tension was measured in interphase and mitotic cells. Moreover, it would be helpful to be more specific about how much higher cortical tension was found to be in mitotic cells.
2. Lines 91-94, it would be helpful to be more specific about what the “ATP regeneration system” is.
3. The images shown in figure 1E aren’t very convincing, it would be helpful if the authors co-stained for F-actin and the plasma membrane. Was the “ATP regeneration system” used before collecting these images? If so, it should be indicated in the figure legend.
4. In figure 4B, the Western blots for septin 9 and GAPDH should be separate in the figure. As it’s shown, it looks as though a single (i.e., uncut) membrane was probed simultaneously with two antibodies.

Discussion:
1. Because the analysis relied upon the isolation of blebs, which inherently require high levels of cortical actomyosin contractility to form, some factors that promote cortical actomyosin contractility may have been missed (i.e., not enriched in blebs from mitotic cells).
2. Perhaps I missed them, but I was surprised that none of the ERM family proteins were identified by mass spectrometry. However, they are detected by Western blot in supplementary figure 1.

Methods:
1. It would be helpful to know if blebs were isolated in the cold.
2. Lines 305-307, it would be more useful to state final concentrations.

<u>Reviewer 2</u>

Advance summary and potential significance to field

In this manuscript, the authors have used mass spectrometry (MS) to profile the proteins that make up the actin cortex in interphase and mitotic cells, in an attempt to identify the proteins that contribute to promoting cortical tension.
Cells were synchronized into the appropriate cell cycle stages, then blebbing was induced in each population with Latrunculin B. Blebs were isolated purified, the actin cortex allowed to re-form within blebs, and proteins were subjected to MS analysis in 3 replicate experiments. In total, 922 proteins were identified in blebs from both treatment groups, of which 238 were related in some way to actin. Septin 9 was selected for more detailed study as a representative protein enriched in the mitotic actin cortex. Knockdown of Septin 9 was found to affect mitotic rounding and circularity, consistent with a previously described role in cytokinesis. The manuscript is a useful catalogue of actin cortex proteins, and a description of changes in their distribution between interphase and mitosis.

The data are convincingly presented and the methodology is clear. The manuscript is logically organized and well written. The information in this resource format manuscript will be of interest to many researchers in cell biology.

© 2022. Published by The Company of Biologists under the terms of the Creative Commons Attribution License (https://creativecommons.org/licenses/by/4.0/).

Comments for the author

The procedures do raise some questions that might be useful to discuss in the manuscript. There are also a few points that should be addressed in a revised manuscript.

1. Why was in necessary to go through a convoluted procedure to induce blebbing with latrunculin and then isolate the blebs? These were not naturally occurring blebs, so it's not clear why this procedure is an advantage over a more straight-forward method for isolating plasma membrane fractions, such as centrifugation using discontinuous sucrose density gradients?
2. It was also not clear if blebs were treated with Hemolysin A and an ATP regeneration system to facilitate cortex reassembly prior to MS analysis? If so was this important for separation of blebs from whole cells? And if it wasn't needed for separation of blebs from whole cells, it's not clear why it would be necessary at all since presumably all the cortical cytoskeleton proteins were contained within the blebs, and there was no further purification of the cytoskeleton.
3. The number of proteins identified in each experimental replicate is somewhat variable as shown in Figure 2, presumably due to differences in the number of spectral counts obtained within each experiment that resulted in differing numbers of statistically significant protein identifications to be made. That being the case, wouldn't it be useful to consider the proportion of each protein's spectral counts out of the total number obtained within each experimental replicate as a way to correct for variations between experiments?
4. Was any principal component analysis performed to look at the variability between experimental replicates? This could be done for all proteins, but would be more interesting in comparing subsets, such as the data in Figure S3. By eye, it is difficult to understand the point of Figure S3, is it to convince the reader of the reproducibility of the experimental replicates? If it's simply of way of depicting the results from Table 1, then a volcano plot would be clearer.
5. One question that study raises is whether most proteins that are apparently more abundant in the mitotic actin cortex are likely due to increased expression, as is the case for Septin 9? It would be beyond the scope of this study, but it would have been interesting to profile total cellular proteins in interphase vs mitotic cells, and then correlate that with the actin-cortex associated proteins to determine which proteins are actually enriched or diminished due to localization versus those that are altered due to changes in cellular levels.
6. The Figure 4C legend says that the panels are from STORM images of the actin cortex, is this correct? The methods don't say anything about STORM imaging but do say that live cell imaging is brightfield.
7. Were the only differences in mitosis in Septin 9 knockdown cells related to the morphology? There were no changes in the timing or completion?
8. Lines 213-214. Cross sections don't seem to be the correct way to measure the changes in area and circularity shown in Figures 4D-E, is that correct? Cross-sections might have been used for the parameters shown in Figure S4D.
9. The Figure legend for Figure indicates panel A, but there is no other panel so this is unnecessary.

<u>Reviewer 3</u>

Advance summary and potential significance to field

This is an interesting proteomic study, which sought to identify cortical proteins associated with the high and low tension of the plasma membrane of mitotic and interphase cells, respectively. The work identifies a number of actin-associated proteins associated with high membrane tension including septin 9.

The proteomic aspects of the work are rigorous and well conducted. The manuscript's data with regard to septin 9 are not as strong, and there are some concerns with regard to the experimental set up used to perform the proteomics.
Membrane tension differences are presumed to be preserved upon a number of treatments, but no experimental data evidence is cited or shown to demonstrate that this is indeed the case.

1. Trypsinization of interphase cells and culture in suspension are expected to affect the tension of interphase adherent cells. The authors cite Chugh et al 2017 for suspension interphase cells having lower tension than mitotic cells that were shaken off. What are the precise differences

© 2022. Published by The Company of Biologists under the terms of the Creative Commons Attribution License (https://creativecommons.org/licenses/by/4.0/).

quantitatively, and how were they measured? How does the membrane tension of adherent interphase cells compare to the membrane tension of adherent mitotic cells? If the differences are not of similar level to the ones observed for and between suspension cells, there are concerns about the physiological significance of this work and approach. This must be addressed with more text or otherwise, even if it's published previously.

2. An additional concern is the use of latrunculin B to generate the blebs which are used for the proteomic analysis. Latrunculin B can also change the cortical tension of both mitotic and interphase cells. How does latrunculin B impact the membrane tension of interphase and mitotic cells? Did the authors make a quantitative measurement of tension upon use of latrunculin B, and demonstrate that tension differences are retained? The same concern pertains the use of hemolysin A. It is unclear if after these treatments, the tension differences are preserved.

3. The authors chose to focus on Sept9, however Sept8 appears to have an even higher ratio of mitotic to interphase spectral counts (3.31 vs 1.63 in Table 1).
In supplemental figure S4A, it is shown that additional septins are upregulated (Sept2, Sept6, Sept7, Sept10, Sept11). However, these are not shown in Table 1.
Why not? Perhaps, a septin-specific table should be made and provided in the main manuscript, with some discussion for the differences seen across the different septins.

It is unclear why the authors decided to focus on Sept9 and did not check how depletion of other septins – especially Sept8 - affects rounding in mitosis. Can the authors perform a co-IP from blebs to determine whether Sept9 functions in complex with the other septins? Or whether there is a distinct Sept9 complex functioning on mitotic membrane of high tension. From a septin standpoint, this would be a very important finding and provide clarity on whether Sept9 functions alone or in complex with other septins.

If a co-IP is not feasible, the authors should check for cortical levels of other septins (by immunofluorescence) upon Sept9 depletion, and perform depletion of other septins from representative groups (e.g., Sept2, Sept6/8, Sept7) to see if they phenocopy Sept9 depletion and if they impact the cortical levels of Sept9.
Lack of an effect will suggest that there are not in a complex, as the depletion of a specific septin is known to impact the levels of its dimeric or complex partners.

4. The data in Figure 4 are not very strong. In the image of Figure 4C, the presumably Sept9-depleted mitotic cell seems to undergo faster cell division (prophase to anaphase A). Rounding appears to be similar with the control during prophase and metaphase, but cells spread out more and faster after cleavage furrow ingression. Sept9 depletion is known to abrogate abscission without affecting prior stages of cell division. Can the reduced rounding be due to the abscission defect?

The authors should perform more of a rigorous analysis of circularity and area in equivalent and different stages of mitosis (prophase, metaphase, anaphase telophase). In addition, they should perform time-lapse experiments with fluorescent markers of the nuclear envelope and/or chromatin (concomitantly with DIC/phase contrast) to determine if there is a delay in the roundness in the early stages of prophase. Lastly, an early loss of roundness in telophase may be indicative of a more efficient spreading rather than a defect in circularity.
Per the comments above, knock-down of some of the other septins should be performed to see if it yields similar phenotype. Or whether this is a Sept9-specific phenotype.

Comments for the author

see above

© 2022. Published by The Company of Biologists under the terms of the Creative Commons Attribution License (https://creativecommons.org/licenses/by/4.0/).

First revision

Author response to reviewers' comments

Reviewer 1 Advance Summary and Potential Significance to Field:
In Vadnjal, N et al., the proteomic composition of the cortical actin network in interphase and mitotic cells is resolved by mass spectrometry. To obtain a pure cortical actin network, which includes numerous associated factors, the authors use a previously devised protocol for isolating blebs. By comparing the cortical actin network in interphase and mitotic cells, the authors identify specific factors required for mitotic cell rounding – a process requiring a heightened level of cortical actomyosin contractility. Thus, factors enriched in blebs from mitotic cells are likely to play a role in this process. Indeed, the authors validate a candidate factor, septin 9, is required for mitotic cell rounding. Due to the involvement of the cortical actin network in numerous processes, this is likely to be a valuable resource to a wide array of cell biologists.

We thank the Reviewer for their assessment of our work and constructive comments.

Reviewer 1 Comments for the Author:
Results:

1. Lines 71-74, as this phenomenon is important to the underlying premise of the article, it would be helpful to be more specific about how cortical tension was measured in interphase and mitotic cells. Moreover, it would be helpful to be more specific about how much higher cortical tension was found to be in mitotic cells.

The protocol for obtaining interphase and mitotic cells, and cortical tension measurements were described in a previous publication from the lab (Chugh et al, Nat Cell Biol 2017). We realised that these points were not introduced clearly enough in our manuscript and have now added further explanations, in particular that cortical tension was measured using atomic force microscopy flat cantilever compression, and is ~4-fold lower in rounded interphase compared to mitotic HeLa cells (lines 86-89).

2. Lines 91-94, it would be helpful to be more specific about what the “ATP regeneration system” is.

The ATP regeneration system is a mixture of ATP, UTP, creatine phosphate and creatine kinase, and is added to allow for cortex reassembly in isolated blebs, as reported in our original paper on bleb isolation (Biro et al, Cytoskeleton 2013). We have now clarified that the ATP-regeneration system is creatine phosphate-based in the text (line 106).

3. The images shown in figure 1E aren’t very convincing, it would be helpful if the authors co-stained for F-actin and the plasma membrane. Was the “ATP regeneration system” used before collecting these images? If so, it should be indicated in the figure legend.

The original Fig.1E was meant to demonstrate that high numbers of small blebs were collected following our bleb isolation procedure. However, we agree that the actin stainings displayed were not making a strong visual point. This is because the blebs displayed in the insets were only a few micrometers in size, and the cortex itself is
~150 nm thick, confocal imaging is thus not sufficient to provide a sharp picture of the cortex in this system. We thus moved the original Fig.1E to supplements (current Fig. S1A) and have replaced it with super-resolution STORM images of exemplar isolated blebs. This provides higher resolution images clearly showing that an actin cortex is re-assembled in isolated blebs. We have also added in the figure legend that the ATP regeneration system was indeed added prior to imaging.

4. In figure 4B, the Western blots for septin 9 and GAPDH should be separate in the figure. As it’s shown, it looks as though a single (i.e., uncut) membrane was probed simultaneously with two antibodies.

The Reviewer’s comment made us realise that it was not clear from the figures that we used fluorescence-based Western blots, where antibodies against septin 9 and GAPDH from different

© 2022. Published by The Company of Biologists under the terms of the Creative Commons Attribution License (https://creativecommons.org/licenses/by/4.0/).

species were used and could thus be imaged together on the same, uncut, membrane. We changed the colour of the Western blots displayed in Fig. 4B from inverted black and white to the original red and green to more clearly represent fluorescent imaging. Of note, for some of the other Western blots in the paper, we used antibodies of the same species for different proteins, and in these cases the membranes were indeed cut (Figs 1C, S1B). We have clarified these points in the legends and have clearly indicated the positions where the membranes were cut, when relevant, in the supplementary figure with uncropped Western blots (Figure blot transparency; arrows point to positions of membrane cutting).

Discussion:

1. Because the analysis relied upon the isolation of blebs, which inherently require high levels of cortical actomyosin contractility to form, some factors that promote cortical actomyosin contractility may have been missed (i.e., not enriched in blebs from mitotic cells).

On the one hand, shearing off blebs detaches them from cells prior to retraction, and might thus lead to under-representation of proteins controlling contractility, which are typically recruited late to the bleb cortex (Charras et al, JCB 2006). However, in our previous paper using the same bleb isolation protocol (Biro et al, Cytoskeleton 2013), we have shown that the isolated blebs displayed active deformations and contractions, indicating that contractility regulators are present in the blebs in sufficient amounts to generate contractile forces. On the other hand, naturally occurring blebs would be favoured by (local or global) high contractility, and might thus be enriched in contractility regulators. However, the rounded interphase cells and prometaphase cells we use are not particularly blebby when unperturbed, likely due to high membrane-to-cortex attachments stability. Thus, in our protocol, bleb formation was induced by the actin depolymerising drug Latrunculin B, which at the concentrations used leads, at first, to local cortex disruption and blebbing that continues for long enough to allow for the resulting blebs to be isolated through mechanical shearing. Thus, as blebs were induced in a generic manner through destabilisation of the actin cortex in both interphase and mitotic cells, we don't expect this protocol to lead to differential enrichment of contractility regulators in different phases of the cell cycle. Importantly, Latruculin was washed out following bleb isolation, an essential step to allow for cortex re-assembly in the isolated blebs. This allowed us to image the cortex in a sample of the isolated blebs prior to mass spectrometry (Figs 1E, S1A). This served as a control to confirm that the isolation worked well and yielded blebs containing all the components required for actin cortex re-assembly. We have clarified these points in the text (lines 107; 288).

2. Perhaps I missed them, but I was surprised that none of the ERM family proteins were identified by mass spectrometry. However, they are detected by Western blot in supplementary figure 1.

In fact, ERM proteins (EZR, RDX, MSN) were detected by mass spectrometry and are listed in Tables S1, S2, S3. Interestingly, for all three members of the ERM family, levels were lower in mitotic blebs compared to interphase blebs; this difference was only significant to moesin (MSN), which is thus the only of the three ERM proteins that appears in Fig. 3 and Table 1. Previous studies from the lab (Chugh et al, Nat Cell Biol 2017) and others (Toyoda et al, Nat Comm 2017) did not observe a strong effect of ERM depletion on cortex organisation or tension in mitosis. However, it is important to note that ERM proteins are activated through phosphorylation, and we have not explored whether phosphorylated ERM levels change between interphase and mitotic blebs. Investigating this question, and further investigating how ERM localisation and activity affects cortex mechanics, will be an interesting avenue for future studies.

Methods:

1. It would be helpful to know if blebs were isolated in the cold.

Cells were maintained at 37°C until bleb induction (Step 2 of the protocol depicted in Fig. 1D), the rest of the detachment protocol was performed at room temperature.
We have now clarified this in the figure legends and methods.

2. Lines 305-307, it would be more useful to state final concentrations.

© 2022. Published by The Company of Biologists under the terms of the Creative Commons Attribution License (https://creativecommons.org/licenses/by/4.0/).

Methods were changed to state final concentrations.

Reviewer 2 Advance Summary and Potential Significance to Field:
In this manuscript, the authors have used mass spectrometry (MS) to profile the proteins that make up the actin cortex in interphase and mitotic cells, in an attempt to identify the proteins that contribute to promoting cortical tension.
Cells were synchronized into the appropriate cell cycle stages, then blebbing was induced in each population with Latrunculin B. Blebs were isolated, purified, the actin cortex allowed to re-form within blebs, and proteins were subjected to MS analysis in 3 replicate experiments. In total, 922 proteins were identified in blebs from both treatment groups, of which 238 were related in some way to actin. Septin 9 was selected for more detailed study as a representative protein enriched in the mitotic actin cortex. Knockdown of Septin 9 was found to affect mitotic rounding and circularity, consistent with a previously described role in cytokinesis. The manuscript is a useful catalogue of actin cortex proteins, and a description of changes in their distribution between interphase and mitosis.

The data are convincingly presented and the methodology is clear. The manuscript is logically organized and well written. The information in this resource format manuscript will be of interest to many researchers in cell biology.

We thank the Reviewer for their assessment of our work and constructive comments.

Reviewer 2 Comments for the Author:

The procedures do raise some questions that might be useful to discuss in the manuscript. There are also a few points that should be addressed in a revised manuscript.

1. Why was in necessary to go through a convoluted procedure to induce blebbing with latrunculin and then isolate the blebs? These were not naturally occurring blebs, so it's not clear why this procedure is an advantage over a more straight-forward method for isolating plasma membrane fractions, such as centrifugation using discontinuous sucrose density gradients?

We had actually tried membrane fraction isolation protocols in the lab, but were not convinced of the purity of the resulting actin cortex preparation. Specifically, we isolated membrane fractions using a "Subcellular Protein fraction kit" (Thermo Fischer, cat. no.: 78840). However, we found it difficult to ascertain that the entire cortex was effectively enriched following this protocol. We have also in the past used Ficoll and sucrose gradients to separate blebs from cells. While this worked well, we preferred using filter-based isolation here to ensure that entire cells were effectively removed from our samples.

In summary, we found that bleb isolation allows for rapid separation of cortical fractions from the rest of the cell with minimal perturbation, taking advantage of a natural phenomenon where the cortex becomes separated from the rest of the cell. Inducing blebs with low doses of Latrunculin is a robust and simple approach, and besides requiring large amounts of starting material, the bleb isolation protocol is in fact relatively straightforward. Previous work from our labs (Biro et al., Cytoskeleton 2013) has shown that bleb isolation represents a reproducible approach to enrich for the actin cortex, and that the cortex reassembled in isolated blebs can display contractions and is structurally similar to cellular actin cortex. Importantly, our approach allowed us to verify that an F-actin cortex was effectively re-assembled in the isolated blebs (Figs 1E, S1A) prior to mass spectrometry. As also discussed in point 2 below, this control confirms that all the regulators required for cortex assembly are retained in the isolated blebs. We have added a short discussion of the advantages of using blebs for cortex enrichment in the discussion section of the revised manuscript.

2. It was also not clear if blebs were treated with Hemolysin A and an ATP regeneration system to facilitate cortex reassembly prior to MS analysis? If so, was this important for separation of blebs from whole cells? And if it wasn't needed for separation of blebs from whole cells, it's not clear why it would be necessary at all since presumably all the cortical cytoskeleton proteins were contained within the blebs, and there was no further purification of the cytoskeleton.

© 2022. Published by The Company of Biologists under the terms of the Creative Commons Attribution License (https://creativecommons.org/licenses/by/4.0/).

As the Reviewer points out, treatment with Haemolysin A and the ATP regeneration system is required for cortex re-assembly, not for the isolation of cortical components. As such, it was indeed not a necessary step for the mass spectrometry analysis. However, we decided to include this step in our protocol, as it allowed us to image the cortex in a sample of the isolated blebs prior to mass spectrometry (Figs 1E, S1A). This served as a control to confirm that the isolation worked well and yielded blebs containing all the components required for actin cortex re-assembly.
We have clarified this in the text.

3. The number of proteins identified in each experimental replicate is somewhat variable as shown in Figure 2, presumably due to differences in the number of spectral counts obtained within each experiment that resulted in differing numbers of statistically significant protein identifications to be made. That being the case, wouldn't it be useful to consider the proportion of each protein's spectral counts out of the total number obtained within each experimental replicate as a way to correct for variations between experiments?

The spectral counts we report were in fact normalised to correct for variations between experiments. Specifically, the total number of spectra identified for each protein was divided by the total spectral count detected in the specific replicate considered, and multiplied by the total spectral count detected in the first interphase replicate, thus normalising for experimental variation between replicates. We have added a sentence explaining this normalisation to the main text and methods.

4. Was any principal component analysis performed to look at the variability between experimental replicates? This could be done for all proteins, but would be more interesting in comparing subsets, such as the data in Figure S3. By eye, it is difficult to understand the point of Figure S3, is it to convince the reader of the reproducibility of the experimental replicates? If it's simply of way of depicting the results from Table 1, then a volcano plot would be clearer.

With Fig. S3 (Fig. S2A in the revised manuscript) we meant to show reproducibility of the experimental replicates for the 54 protein candidates listed in Table 1, as well as provide information about the levels of each protein in each of the replicates. We agree that this figure is somewhat had to read. Thus, as per Reviewer's suggestion, we have now tested alternative representations of reproducibility. We compared the levels of the proteins listed in Table 1 in the different replicates from interphase and mitotic cortices using PCA (see Reviewer Figure below) and a heatmap representation (new Fig. S2B). Both analyses show that the interphase and mitotic replicates cluster separately, highlighting the reproducibility of the experimental replicates. Interphase replicate 3 differs from the other two interphase replicates along Principal Component (PC) 2, but PC2 represents only ~16% of the variability between the samples. Interphase replicates cluster together along PC1, which represents over 70% of the variability between the samples.

© 2022. Published by The Company of Biologists under the terms of the Creative Commons Attribution License (https://creativecommons.org/licenses/by/4.0/).

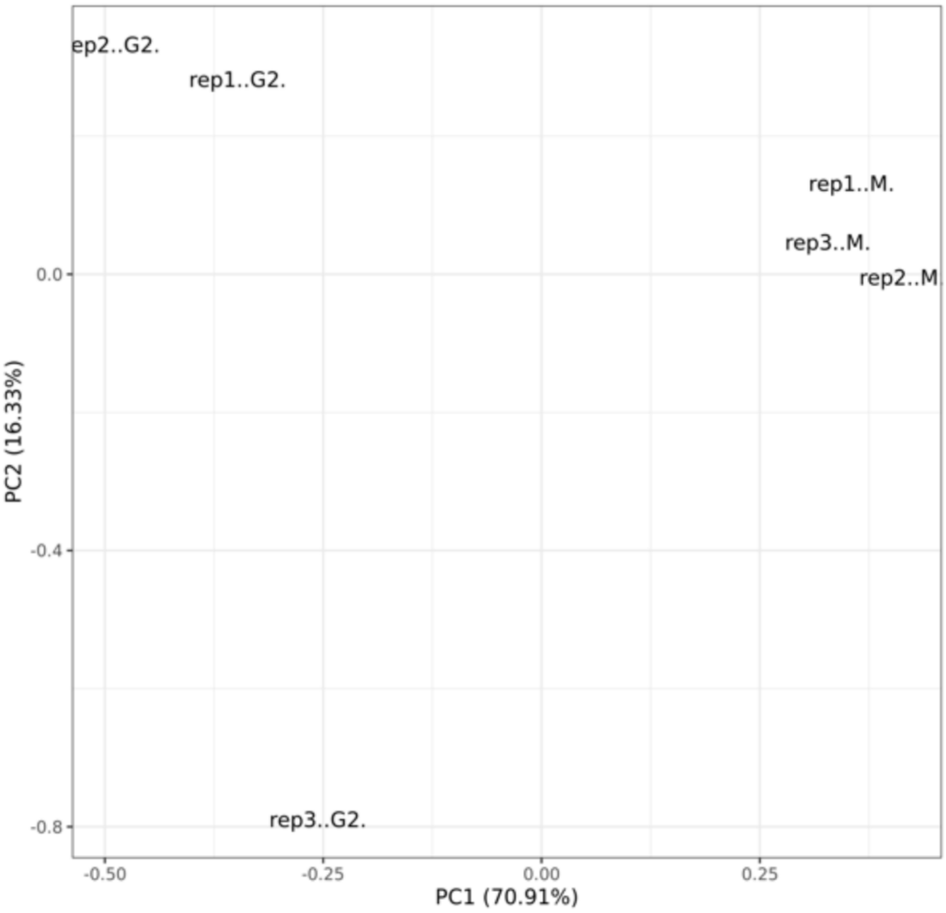

Reviewer Figure 1: Principal component analysis of mass spectrometry data (for the 54 actin related proteins listed in Table 1). The analysis shows that the mitotic replicates (rep 1, 2, and 3 M) cluster together, and separately from the interphase replicates (rep 1, 2, 3 G2).

5. One question that study raises is whether most proteins that are apparently more abundant in the mitotic actin cortex are likely due to increased expression, as is the case for Septin 9? It would be beyond the scope of this study, but it would have been interesting to profile total cellular proteins in interphase vs mitotic cells, and then correlate that with the actin-cortex associated proteins to determine which proteins are actually enriched or diminished due to localization versus those that are altered due to changes in cellular levels.

To address the Reviewer's comment, we have now performed this analysis for the 54 proteins listed in Table 1, using a published dataset for total cellular protein levels. Specifically, we compared differences in protein levels detected in blebs to differences in protein levels between interphase and mitosis from a mass spectrometry analysis of entire HeLa cells (as reported in Heusel et al Cell Syst 2020, who used a synchronisation protocol comparable, though slightly different from our study). Comparison of actin related protein levels in blebs and whole cells showed some interesting differences in enrichment, now shown in new Fig. S2C. Notably, while most of the proteins that increased in mitotic blebs also increased in cells, for most proteins the extent of the increase was higher in blebs than in cells.
Furthermore, most proteins that showed decreased levels in mitotic blebs, displayed increased levels in mitotic cells. Together, these observations suggest that differences in cortical composition between interphase and mitosis are not only the result of differences in expression levels but that cortical recruitment is tightly regulated.

6. The Figure 4C legend says that the panels are from STORM images of the actin cortex, is this correct? The methods don't say anything about STORM imaging but do say that live cell imaging is brightfield.

© 2022. Published by The Company of Biologists under the terms of the Creative Commons Attribution License (https://creativecommons.org/licenses/by/4.0/).

We thank the reviewer for pointing out the mistake, which was now corrected. Now the figure legend accurately describes images as brightfield and not STORM. We have also now added STORM pictures of isolated blebs in Fig. 1E, as they provide a better visualisation of the actin cortex (see also our answer to comment 3 from Reviewer 1).

7. Were the only differences in mitosis in Septin 9 knockdown cells related to the morphology? There were no changes in the timing or completion?

We have now quantified the timing of rounding and ingression in septin 9-depleted cells (new Fig. S3D). We found that septin 9 depletion slightly increased rounding time but did not significantly affect ingression time. Together with the rounding defects we report, these observations support a role for septin 9 in the control of cell surface mechanics in early stages of mitosis. Additionally, following suggestions from Reviewer 3, to further explore the role of septins in mitotic cell shape control, we have performed additional experiments investigating the effects of depletion of three other septins from different septin subgroups, septin 2, septin 7 and septin 8 (new Fig. S4). We show that depletion of these septins affects cell circularity in metaphase. Together, our data strongly support an important role for septins in cell surface mechanics in early stages of mitosis.

8. Lines 213-214. Cross sections don't seem to be the correct way to measure the changes in area and circularity shown in Figures 4D-E, is that
correct? Cross-sections might have been used for the parameters shown in Figure S4D.

The comment made us realise our phrasing was mis-leading: area and circularity were analysed in mid-plane images of the cells, so in x-y cross-sections (not vertical cross-sections). For quantification of all cell shape parameters (reported in current Figs 4D, E, S3C S4B, S4C, S4D), midplanes of metaphase cells, as shown in Figs 4C and S3B, were used in order to analyse rounding defects in the cell's equatorial plane. We have corrected the text to clarify how our measurements were performed, and have added additional pictures of cellular equatorial mid-planes (Fig. S3B).

9. The Figure legend for Figure indicates panel A, but there is no other panel so this is unnecessary.

We assume the Reviewer referred to Fig. S3 (Fig. S2 in revised manuscript), which was indeed single panel in our original submission. This Figure has been changed in the revised manuscript and now contains multiple panels.

Reviewer 3 Advance Summary and Potential Significance to Field:
This is an interesting proteomic study, which sought to identify cortical proteins associated with the high and low tension of the plasma membrane of mitotic and interphase cells, respectively. The work identifies a number of actin-associated proteins associated with high membrane tension including septin 9.

The proteomic aspects of the work are rigorous and well conducted. The manuscript's data with regard to septin 9 are not as strong, and there are some concerns with regard to the experimental set up used to perform the proteomics. Membrane tension differences are presumed to be preserved upon a number of treatments, but no experimental data evidence is cited or shown to demonstrate that this is indeed the case.

We thank the Reviewer for their assessment of our work and constructive comments.

1. Trypsinization of interphase cells and culture in suspension are expected to affect the tension of interphase adherent cells. The authors cite Chugh et al 2017 for suspension interphase cells having lower tension than mitotic cells that were shaken off. What are the precise differences quantitatively, and how were they measured? How does the membrane tension of adherent interphase cells compare to the membrane tension of adherent mitotic cells? If the differences are not of similar level to the ones observed for and between suspension cells, there are concerns about the physiological significance of this work and approach. This must be addressed with more text or otherwise, even if it's published previously.

© 2022. Published by The Company of Biologists under the terms of the Creative Commons Attribution License (https://creativecommons.org/licenses/by/4.0/).

The Reviewer's comment made us realise we had not been sufficiently clear in explaining the rationale for our choice of experimental system, and we have now added further explanations in the manuscript. Our study aims to compare cortical compositions between cells with high and low cortical tensions. We used HeLa cells in interphase and mitosis as a model system because they have become an experimental system of choice for studies of cortical tension regulation (see for instance Toyoda et al Nat Comm 2017 and Chugh et al. Nat Cell Biol 2017, all cited in the paper). Indeed, it has been previously shown by several labs, including ours, that cortical tension increases upon mitosis entry in HeLa cells (see for instance Stewart et al Nature 2011, Chugh et al Nat Cell Biol 2017, Ramanathan et al Nat Cell Biol 2015). Importantly, we focus on cortical tension, as measured by AFM flat cantilever compression, which controls cellular rounding, and not membrane tension, which is a measure of the plasma membrane resistance to deformation (and is typically measured by pulling membrane tubes from the cell surface with e.g. an optical tweezer). We have clarified throughout the text the tension we refer to is cortical tension. Physiologically, the cortical tension increase is important for mitotic progression, as it promotes mitotic cell rounding, which in turn facilitates correct spindle assembly and metaphase plate formation (as discussed in Lancaster and Baum Semin. Cell Dev. Biol 2014). The extent of the tension increase depends on the exact subline of HeLa cells used. With the subline we use here, HeLa TDS, we have previously shown that detached interphase cells display a cortical tension over 4 times lower than mitotic cells (Chugh et al Nat Cell Biol 2017). This difference is comparable to the tension difference between (detached) interphase and mitotic HeLa Kyoto cells cortex tension (Serres et al Dev Cell 2020; this is also the sub-line used in Toyoda et al Nat Comm 2017), and is higher than the tension difference displayed between interphase and mitosis by S-HeLa cells, a subline of HeLa cells that is continuously cultured in suspension (Chugh et al Nat Cell Biol 2017). Finally, we focused on interphase detached cells rather than adherent cells because we wanted to compare cortices. Adherent interphase HeLa cells are spread and while an actin cortex is present at the top of the cells, actin organisation is dominated by lamellipodia and filopodia. They would thus not be an appropriate system for comparing cortices with low and high tension.

We have now added information on how cortical tension was measured, along with a clarification that the cells were synchronised and rounded up following the same protocol as Chugh et al Nat Cell Biol 2017, which reported clear differences in cortical tension. In summary, we have now clarified that detached interphase cells are used here as a model system for cortices with lower tension than in mitotic cells. We believe that this is a physiologically relevant approach, as the interphase cells are used as a reference point to identify cortical proteins potentially involved in the regulation of cortical tension, and high cortical tension is in turn essential for correct mitosis progression.

2. An additional concern is the use of latrunculin B to generate the blebs, which are used for the proteomic analysis. Latrunculin B can also change the cortical tension of both mitotic and interphase cells. How does latrunculin B impact the membrane tension of interphase and mitotic cells? Did the authors make a quantitative measurement of tension upon use of latrunculin B, and demonstrate that tension differences are retained? The same concern pertains the use of hemolysin A. It is unclear if after these treatments, the tension differences are preserved.

Latrunculin B was used only to induce blebbing and was washed out after 15 min; the rest of the bleb isolation protocol (from Step 4 Fig. 1D, we have now clarified this in the figure) was performed in the absence of Latrunculin B. Importantly, Latrunculin's action on actin has been shown to be quickly reversible (Mseka et al. J Cell Sci 2007), as also discussed in our original paper on bleb isolation (Biro et a;l., Cytosksleton 2013). Similarly, Haemolysin A was only added for a limited time to permeabilise the bleb membrane and allow for the uptake of the ATP regeneration system, which is important for reassembly of a dynamic actin cortex in blebs. Both Haemolysin A and the ATP regeneration system were washed off prior to mass spectrometry; we have now clarified this in the Methods. Strictly speaking, as pointed out by Reviewer 2, cortex reassembly was not required in our experiment, as all the proteins detected in the mass spectrometry are present in the isolated blebs prior to cortex reassembly. However, inducing cortex reassembly through addition of the ATP regeneration system allowed us to image the cortex in a sample of the isolated blebs prior to mass spectrometry (Fig. S1A). This served as a control to confirm that the isolation worked well and yielded blebs containing all the components required for actin cortex re-assembly. We have clarified these points in the text.

© 2022. Published by The Company of Biologists under the terms of the Creative Commons Attribution License (https://creativecommons.org/licenses/by/4.0/).

We had previously shown that blebs isolated using a similar protocol display cortices similar to the cellular cortex, as imaged with confocal and electron microscopy (Biro et al Cytoskeleton 2013). Furthermore, we observed that blebs isolated from interphase cells were generally less round than those isolated from mitotic cells (as suggested in the images in new Fig. 1E); this suggests a lower surface tension in interphase blebs compared to mitotic blebs, similarly to what is observed in entire cells. We have attempted to measure cortical tension of isolated blebs using the AFM protocol we use for measuring cortical tension in cells. This approach relies on precisely measuring cell deformation upon compression under an AFM cantilever (see for instance Chugh et al Nat Cell Biol 2017). Unfortunately, we found that the technique cannot be directly used to measure tension of isolated blebs, as compression was difficult to achieve in most cases due to the small size of the blebs (< 5 μm in diameter) and the slight angle between the cantilever and the substrate: this made exerting compression with a flat cantilever challenging. Directly measuring tension in isolated blebs is an exciting question, which will require technological development and will be the subject of a follow up study of bleb mechanics in the lab. We have added a short discussion of these points in the Discussion section of the paper.

3. The authors chose to focus on Sept9, however Sept8 appears to have an even higher ratio of mitotic to interphase spectral counts (3.31 vs 1.63 in Table 1).
In supplemental figure S4A, it is shown that additional septins are upregulated (Sept2, Sept6, Sept7, Sept10, Sept11). However, these are not shown in Table 1. Why not? Perhaps, a septin-specific table should be made and provided in the main manuscript, with some discussion for the differences seen across the different septins.

Following the Reviewer's comments, we have now moved the graph reporting changes in levels for all the septins detected in blebs (Fig. S4A in the original submission) to a main figure (current Fig. 4A), and have added the related P-values in the figure legend. Even though, as pointed out by the Reviewer, several septins appear to be changing levels between interphase and mitosis, these changes were statistically significant only for septin 9 and septin 8. This is why these are the only members of the septin family included in Table 1, which lists actin-related proteins displaying a statistically significant (P-value < 0.05) change in levels between interphase and mitosis.

In our original submission, we had focused on the role of septin 9 in mitotic rounding because it was significantly enriched in mitotic blebs and it had previously been shown to affect the mechanics of mitotic cells (Toyoda et al. Nat Comm 2017).
Furthermore, it was the only member of its group (SEPT3 group, which includes septins 3, 9 and 12) detected in blebs, which limited the risk of redundancy and compensation by other septins upon depletion. To address the Reviewer's comments, we have now complemented this investigation and tested the effects of depleting several other septins, as described below.

It is unclear why the authors decided to focus on Sept9 and did not check how depletion of other septins – especially Sept8 - affects rounding in mitosis. Can the authors perform a co-IP from blebs to determine whether Sept9 functions in complex with the other septins? Or whether there is a distinct Sept9 complex functioning on mitotic membrane of high tension. From a septin standpoint, this would be a very important finding and provide clarity on whether Sept9 functions alone or in complex with other septins.

If a co-IP is not feasible, the authors should check for cortical levels of other septins (by immunofluorescence) upon Sept9 depletion, and perform depletion of other septins from representative groups (e.g., Sept2, Sept6/8, Sept7) to see if they phenocopy Sept9 depletion and if they impact the cortical levels of Sept9.
Lack of an effect will suggest that there are not in a complex, as the depletion of a specific septin is known to impact the levels of its dimeric or complex partners.

We have performed new experiments to address the Reviewer's concerns. Specifically, we investigated whether other septins also play a role in mitotic rounding, and assessed whether depletion of specific septins affects the expression levels of other septins. We decided to deplete one septin from each of the four major septin homology groups (SEPT2, 3, 6 and 7). Septin 9 was the only member of the SEPT 3 group detected in our isolated bleb fractions, and was investigated in our original submission. We now also depleted septin 2 and septin 7, the only members of SEPT2 and SEPT7 groups detected in blebs, and septin 8, the member of the SEPT6 group displaying the

© 2022. Published by The Company of Biologists under the terms of the Creative Commons Attribution License (https://creativecommons.org/licenses/by/4.0/).

most significant change in levels between interphase and mitotic blebs (the other members of the SEPT6 group detected in blebs are septin 6, septin 10 and septin 11). We then quantified the effects on mitotic cell shape and division dynamics (new Fig. S4). We found that depletion of all three septins led to decreased circularity of mitotic cells, indicating that alongside septin 9, several other septins regulate mitotic rounding. Furthermore, while septin 9 depletion also resulted in a slightly increased rounding time (new data in Fig. S3D), we found no significant change in rounding time upon depletion of septin 2, septin 7 or septin 8. Finally, we assessed using qPCR whether depletion of specific septins affected expression levels of other septins (new Fig. S4A). Even though slight changes in levels of some of the septins were detected, these changes were small and did not suggest any clear pattern of compensation upon depletion of any specific septin. Taken together, our new results strengthen our conclusion that septins play an important role in the regulation of cell shape mechanics in early mitosis.

Regarding localisation, we had previously attempted to image several of the septins identified in blebs using commercially available antibodies but could not obtain stainings of high enough quality, possibly due to non-specific binding. A previous study (Estey et al J Cell Biol 2010) using custom-made antibodies has shown that septin 2, septin 9, and possibly septin 7 display cortical localisation in early mitosis; we cited this paper in our original submission to comment on septin 9 localisation and have now extended the discussion to comment on the localisation of other septins (septin 2 and septin 7). We believe that a full investigation of how different septins act together to modulate cortex mechanics in early mitosis is beyond the scope of our Resource paper and should be the object of a dedicated project.
Nonetheless, our new data suggest that multiple septins contribute to cell shape regulation in early mitosis.

4. The data in Figure 4 are not very strong. In the image of Figure 4C, the presumably Sept9-depleted mitotic cell seems to undergo faster cell division (prophase to anaphase A). Rounding appears to be similar with the control during prophase and metaphase, but cells spread out more and faster after cleavage furrow ingression. Sept9 depletion is known to abrogate abscission without affecting prior stages of cell division. Can the reduced rounding be due to the abscission defect?

To address the Reviewer’s comment, we have now quantified the duration of mitotic rounding (from the onset of rounding, when cell edge retraction is first observed, to either a stably rounded shape, or to the onset of cytokinesis if active cell rounding continues through metaphase, see details in Methods) and ingression time (from the last frame where aligned chromosomes were observed until the end of cleavage furrow ingression). These quantifications, reported in the new Fig. S3D, show that depletion of septin 9 slightly increases rounding time, but does not significantly affect ingression time. Together with the measurements from (Toyoda et al Nat Comm 2017), this supports our conclusion that septin 9 depletion affects the changes in cell surface mechanics that lead to mitotic rounding, one of the first events of mitosis.
The reported effects on abscission occur much later, after cytokinetic furrow ingression, and would thus not be expected to affect the early mitosis rounding stage.

We realised the timelapse displayed in the original Fig. 4C was confusing, therefore we decided to perform quantifications of the rounding and ingression times, as described above. We now include representative still images of mitotic cells in Fig. 4C, S3B, while the dynamics of cell division are better represented in supplementary videos (Movies S1, S2) and related quantifications (Fig S3D).

The authors should perform more of a rigorous analysis of circularity and area in equivalent and different stages of mitosis (prophase, metaphase, anaphase, telophase). In addition, they should perform time-lapse experiments with fluorescent markers of the nuclear envelope and/or chromatin (concomitantly with DIC/phase contrast) to determine if there is a delay in the roundness in the early stages of prophase.

The aim of quantification of cell shape was to determine whether the depletion of a candidate protein (septin 9 in the original submission, and other members of the septin family as described above) identified in our mass spectrometry analysis of interphase and mitotic cortices, affects cortex-driven mitotic cell shapes. We decided to focus on metaphase because cortex tension is highest during metaphase (see for instance Fischer-Friedrich et al. Sci Rep 2014), we thus reasoned

© 2022. Published by The Company of Biologists under the terms of the Creative Commons Attribution License (https://creativecommons.org/licenses/by/4.0/).

that defects related to mis-regulation of the mitotic cortex will be clearest in the stage of cell division. We found that brightfield images allowed for clear visualisation of chromosomes, which was sufficient for the identification of metaphase and of anaphase onset. To address the Reviewer's comment, we have now added quantifications of the duration of mitotic rounding and of the duration of cytokinetic ingression, as explained above (new Fig. S3D for septin 9 and Fig. S4B-D for septins 2, 7 and 8). We believe that these new quantifications provide a more complete picture of how septin depletion affects various cortex-driven mitotic shape changes.

Lastly, an early loss of roundness in telophase may be indicative of a more efficient spreading rather than a defect in circularity.

As highlighted by the Reviewer, we aimed to focus on cortex-driven cell shape changes, rather than shape changes resulting from e.g. cell spreading at the end of cell division. For this reason, we decided to focus on the early stages of mitosis and quantified cellular shape in metaphase. Our new quantifications of division dynamics (Figs. S3D and S4B-D) further assess cell rounding time, which also focuses on cell shape changes prior to cytokinesis. We have also quantified ingression time, which includes the early stages of telophase (Fig. S3D and S4B-D) and found no significant change following septin depletion. This further supports our conclusions that septins affect cortex mechanics in early stages of mitosis.

Per the comments above, knock-down of some of the other septins should be performed to see if it yields similar phenotype. Or whether this is a Sept9-specific phenotype.

As per Reviewer's suggestion we have now depleted septin 2, septin 7 and septin 8 and assessed the effects on mitotic cell shape, as well as rounding and ingression times, as discussed above and in our response to Point 3. These new data are reported in Fig. S4 and discussed on p. 13 of the revised text.

Second decision letter

MS ID#: JOCES/2022/259993

MS TITLE: Proteomic analysis of the actin cortex in interphase and mitosis

AUTHORS: Neza Vadnjal, Sami Nourreddine, Genevieve Lavoie, Murielle Serres, Philippe P Roux, and Ewa K Paluch
ARTICLE TYPE: Tools and Resources

I am happy to tell you that your manuscript has been accepted for publication in Journal of Cell Science, pending standard ethics checks.

© 2022. Published by The Company of Biologists under the terms of the Creative Commons Attribution License (https://creativecommons.org/licenses/by/4.0/).
